# Wireless Magnetic Robot for Precise Hierarchical Control of Tissue Deformation

**DOI:** 10.1002/advs.202308619

**Published:** 2024-07-23

**Authors:** Chao Wang, Zhi Zhao, Joonsu Han, Arvin Ardebili Sharma, Hua Wang, Xiaojia Shelly Zhang

**Affiliations:** ^1^ Department of Civil and Environmental Engineering University of Illinois Urbana‐Champaign Urbana IL 61801 USA; ^2^ Department of Materials Science and Engineering University of Illinois Urbana‐Champaign Urbana IL 61801 USA; ^3^ Department of Mechanical Science and Engineering University of Illinois Urbana‐Champaign Urbana IL 61801 USA; ^4^ National Center for Supercomputing Applications University of Illinois Urbana‐Champaign Urbana IL 61801 USA

**Keywords:** conttopology optimization, ex vivo application, mechanotherapy, tissue deformation, wireless magnetic robot with remote control

## Abstract

Mechanotherapy has emerged as a promising treatment for tissue injury. However, existing robots for mechanotherapy are often designed on intuition, lack remote and wireless control, and have limited motion modes. Herein, through topology optimization and hybrid fabrication, wireless magneto‐active soft robots are created that can achieve various modes of programmatic deformations under remote magnetic actuation and apply mechanical forces to tissues in a precise and predictable manner. These soft robots can quickly and wirelessly deform under magnetic actuation and are able to deliver compressing, stretching, shearing, and multimodal forces to the surrounding tissues. The design framework considers the hierarchical tissue‐robot interaction and, therefore, can design customized soft robots for different types of tissues with varied mechanical properties. It is shown that these customized robots with different programmable motions can induce precise deformations of porcine muscle, liver, and heart tissues with excellent durability. The soft robots, the underlying design principles, and the fabrication approach provide a new avenue for developing next‐generation mechanotherapy.

## Graphical abstract

Wireless magneto‐active soft robots are developed to achieve various modes of deformations under magnetic actuation and apply forces to tissues in a predictable manner. The design framework considers the hierarchical tissue‐robot interaction and designs customized robots for tissues with varied mechanical properties. The soft robots, design principles, and fabrication approach provide a new avenue for developing next‐generation mechanotherapy.

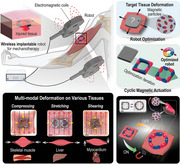

## 1 Introduction

**Figure 1:**
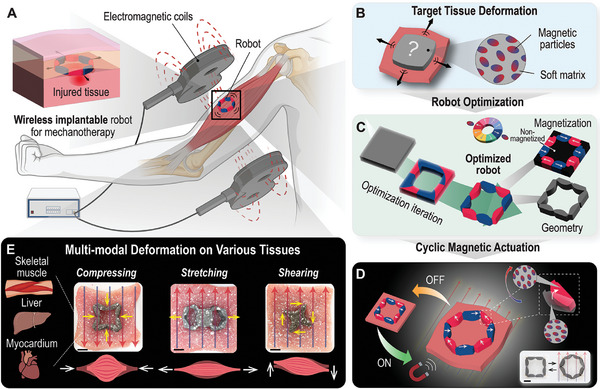
Design principle and application of the precise hierarchical control strategy of the magneto‐active robots. A) A schematic illustrating the potential application of the wireless implantable magneto‐active soft robot and its actuation mechanism to generate mechanical stimulation to tissues. B) Design setup and the target tissue deformation. C) Topology optimization of the robot's geometry and magnetization distribution. D) Cyclic magnetic actuation of the optimized magneto‐active robot. The subplot demonstrates the fabricated robot under cyclic magnetic actuation. E) Ex vivo experiments of the fabricated robots programmed with multi‐modal deformation and applications on different types of porcine tissues: skeletal muscle, liver, and myocardium. The complete procedure of inverse design, hybrid fabrication, mechanical performance test, and ex vivo application is described in Movie S1 (Supporting Information). Scale bar: 10 mm. Schematic diagram in (A) was created with BioRender.com.

Mechanotherapy, which functions by applying mechanical forces to injured or diseased tissues, is a promising treatment option for tissue repair and rehabilitation (**Figure** **1**A). Recent advances^[^ ^1^, ^2^, ^3^, ^4^, ^5^, ^6^, ^7^, ^8^, ^9^ ^]^ have uncovered the ability of mechanical stimuli to regulate cell proliferation, cell differentiation, and inflammatory responses, thereby facilitating the restoration of injured tissues. One main challenge, though, is the development of advanced actuation systems capable of generating controllable forces or strains toward the target tissues. Bulky robotic devices equipped with real‐time force control were able to apply cyclic compressive loading to tissues and improve the recovery of injured leg muscles in a mouse model.^[^ ^6^ ^]^ However, the bulkiness and complexity of these robots limit their widespread use, and pose a concern on the controllability of forces that can be applied to a certain small region of tissues and tissue microenvironment. Moreover, these robots could only be used to stimulate surface tissues. More recently, a mechanically active gel–elastomer–nitinol tissue adhesive (MAGENTA) that can be implanted over tissues was developed to generate forces toward the underlying tissues.^[^ ^10^ ^]^ While this device was able to stimulate muscles with a desired strength and attenuate muscle atrophy, the necessity of embedding an electrical wire and utilizing electricity to generate heat to contract the thermoresponsive nitinol and generate forces poses a concern for practical use.

Moving forward, the development of advanced actuation systems that are 1) biocompatible and easily scalable; 2) capable of achieving precise programmable, controllable, and various modes of loading to deep tissues; and 3) feasible for remote and wireless control will facilitate systematic and controlled preclinical and clinical studies.^[^ ^11^, ^12^ ^]^ While significant progress has been made in the field of soft robotics in generating mechanical actuation,^[^ ^13^, ^14^, ^15^ ^]^ many mechanisms lack wireless or remote control and are not suitable for biomedical applications. One promising candidate is the hard‐magentic soft material.^[^ ^16^, ^17^, ^18^, ^19^ ^]^ These materials consist of soft elastomeric matrices embedded with hard magnetic particles. When magnetically saturated, they can maintain a high remanent magnetization, enabling rapid, untethered, and reversible shape transformations under magnetic actuation.^[^ ^20^, ^21^ ^]^ The external magnetic field can be wirelessly applied and controlled, making hard‐magnetic soft materials an excellent choice for developing functional actuators for biomedical purposes.^[^ ^22^, ^23^, ^24^, ^25^, ^26^ ^]^ In parallel, topology optimization,^[^ ^27^, ^28^, ^29^ ^]^ an effective approach for designing materials and structures to satisfy specific objectives within given constraints, has demonstrated its power in programming various actuation modes of hard‐magnetic soft materials in a controlled manner.^[^ ^30^, ^31^, ^32^, ^33^ ^]^

Leveraging the hard‐magnetic soft material and the multiphysics topology optimization approach, here we report the development of wireless magneto‐active soft robots that can mechanically stimulate and deform various tissues (skeletal muscle, liver, and myocardium) in a wireless, programmable, and precise manner (Figure 1). The robots, which can easily adhere to tissues, can also induce different motion modes (compressing, stretching, and shearing) of the underlying tissues, all in a predictable and precise manner. Our topology optimization approach takes into account the complex mechanical properties of both tissues and robotic materials, as well as their hierarchical interactions, and thus allows for custom‐design of robots for different types of tissues. Robots are fabricated via a facile and biocompatible mold‐casting approach. We show that the fabricated robots display as‐designed motion modes and speeds, with or without attachment to tissues, in the presence of magnetic actuation. By switching on and off the magnetic field, cyclic mechanical loading with robust durability can also be achieved. The complete procedure of inverse design, hybrid fabrication, mechanical performance test, and ex vivo application of the wireless magnetic robot is described in Movie S1 (Supporting Information).

## 2 Results

### 2.1 Biomaterial Robot Design, Fabrication, Characterization, and Biocompatibility Evaluation

The inverse design paradigm and validation process of the biomaterial robot are depicted in Figure 1. The design setup is illustrated in Figure 1A,B. We first define a design domain in space for the robot, which will be positioned on the injured tissue surface. Under magnetic actuation, the movement of the magnetic‐responsive robots triggers the movement of the underlying tissue (robots tightly adhere to the tissue). Then, we specify target tissue deformations (Figure 1B) serving as the objective of our topology optimization framework. Figure 1C illustrates the optimization process. In this study, we incorporate two sets of design variables within the topology optimization framework: the biomaterial robot geometry represented by the density variable distribution and the remanent magnetization distribution (The color wheel in Figure 1C represents the 8 candidate magnetization vectors with the same magnitude but in different directions). Given the target tissue deformation and considering the mechanical properties of both the tissue and the biomaterial, the topology optimization framework optimizes the robot's geometry and magnetization. This process results in the generation of the final optimized robotic design, as illustrated in Figure 1C. When applying the magnetic field (Figure 1D), the magnetic‐responsive robot aligns with the applied magnetic field direction through magnetic torque under wireless and remote control, generating robot motion and inducing tissue deformation. Once the optimized designs are obtained, we conduct ex vivo tests on various porcine tissues (Figure 1E), including skeletal muscle, liver, and myocardium, to verify the actuation performance and durability of the robots.

**Figure 2:**
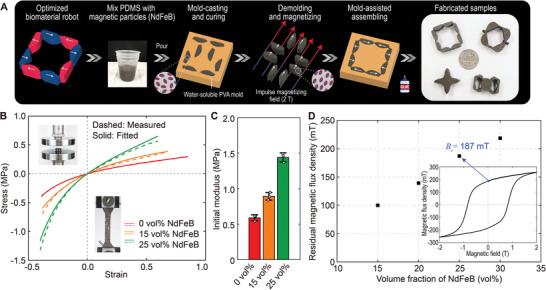
Material synthesis and characterization. A) Fabrication process of the optimized designs. B) Stress–strain relationship and C) initial Young's modulus for PDMS (20:1 base‐to‐agent ratio) with 0 vol%, 15 vol%, and 25 vol% NdFeB magnetic particles. (The data are presented as mean ± SD for *n* = 3 measurements.) D) Measured residual magnetic flux density for material with different volume fractions of magnetic particles. The inset shows the magnetization hysteresis loop for the sample with 25 vol% NdFeB magnetic particles.

**Figure** **2**A illustrates the fabrication process of the optimized biomaterial robots through a molding and casting approach (refer to Experimental Section for detailed information). Given the optimized design, the material is fabricated by mixing polydimethylsiloxane (PDMS) elastomer (with a base‐to‐agent ratio of 20:1) with neodymium‐iron‐boron (NdFeB) hard magnetic particles, and pouring the mixture into 3D‐printed polyvinyl alcohol (PVA) molds for curing at 80 °C. Note that the magnetic particles remain unmagnetized at this step. After the mixture is fully cured, the material components are removed from the molds. An impulse magnetizing field (2 T) is then applied to the cured material to magnetize the embedded NdFeB microparticles in the designated magnetization directions. These individual components are then assembled into a single integrated robot. Figure 2A showcases the library of fabricated samples utilized in this study.

To model the nonlinear magneto‐mechanical performance of robots, we next characterize the mechanical and magnetic properties of PDMS elastomers embeded with 0 vol%, 15 vol%, and 25 vol% NdFeB magnetic particles, respectively. As shown in Figure 2B, the stress–strain curves demonstrate an increase in stiffness as the volume fraction of embedded magnetic particles increases. This stiffening effect is caused by the hydrodynamic effect provided by the addition of the filled rigid magnetic particles.^[^ ^34^ ^]^ To characterize the nonlinear mechanical behavior of the materials, we employ an *I*_1_‐based hyperelastic model^[^ ^35^ ^]^ to fit the measured data (Section S3 and Figure S2, Supporting Information). Figure 2C shows the initial modulus of the three elastomers. We can see the fitted stress–strain curves match well with the measured data. By precisely characterizing the material property, we can accurately predict the nonlinear elastic responses of the material under large deformation and arbitrary stimulation cases, which is crucial for the design and optimization of the soft robots. Understanding the stiffening effect of the hard magnetic soft material with different volume fractions of filled particles allows us to tailor the robot's stiffness for various tissue applications. This also enables the capability of multi‐material design to facilitate more complex functionalities. To capture the magnetic properties of the robots, we measure the residual magnetic flux density $B_{r}$ for the three elastomers. The results (Figure 2D) reveal a nearly linear correlation between the measured $B_{r}$ and the volume fraction of magnetic particles. The inset presents the magnetization hysteresis loop for the sample with 25 vol% magnetic particles, resulting in a residual magnetic flux density of $B_{r}=187$ mT. A higher $B_{r}$ can induce a larger actuated deformation, which is essential for generating sufficient mechanical stimuli to the tissue. Therefore, to ensure the robot's efficacy in generating the desired mechanical response while also considering manufacturability (as achieving a uniform mixing becomes more challenging with higher volume fractions of magnetic particles), we utilize the 25 vol% particle concentration for fabrication and subsequent experiments. These characterized results, aligning within a reasonable range compared to the literature,^[^ ^22^, ^36^, ^37^ ^]^ provide important insights into the mechanical behavior of the synthetic biomaterials, which are essential for the design, simulation, and experimental validation for the optimized robots. The details of the material characterization procedures and constitutive modeling are described in the Supporting Information.

**Figure 3:**
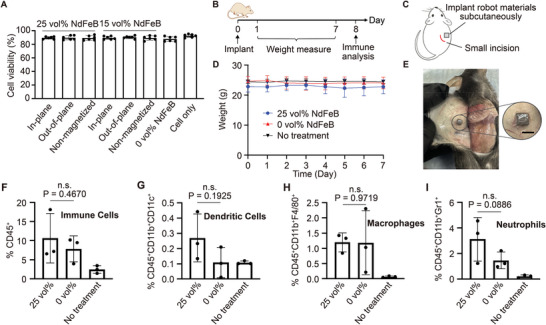
In vitro and in vivo biocompatibility tests of the biomaterial. A) Quantification of cell viability after cultured on the biomaterials for 72 h (*n* = 6). B) Timeline of in vivo biocompatibility (*n* = 3 for each group). On day 0, a small incision on the back skin of immunocompetent C57BL/6 mice was cut, and PDMS containing 25 vol% NdFeB magnetic particles or pure PDMS (3 mm × 3 mm × 1 mm) was placed in the subcutaneous pocket. The body weight of mice was closely monitored and immune cell analyses of skin tissues surrounding the implants were performed on day 8. C) Schematic illustration of implantation. D) Body weight of mice over time. E) Picture of mouse at 8 days post implantation of PDMS containing 25 vol% NdFeB magnetic particles. Scale bar: 3 mm. F) Percentages of CD45^+^ immune cells in the implant area. G) Percentages of CD45^+^CD11b^+^CD11c^+^ dendritic cells in the implant area. H) Percentages of CD45^+^CD11b^+^F4/80^+^ macrophages in the implant area. I) Percentages of CD45^+^CD11b^+^Gr1^+^ neutrophils in the implant area. All the data are presented as mean ± SD. The results are deemed significant at 0.01 < *p ⩽ 0.05, highly significant at 0.001 < **p ⩽ 0.01, and extremely significant at ***p ⩽ 0.001. (n.s., non‐significant.).

To evaluate the biocompatibility of the biomaterial in vitro, 3T3‐L1 fibroblasts are cultured with the material of varied volume fractions (0%, 15%, and 25%) of magnetic particles and varied magnetization directions (in‐plane, out‐of‐plane, and non‐magnetized) for 72 h. As shown in **Figure** **3**A, cells in all groups show good viability, and negligible differences are observed between the robot and non‐treated groups, demonstrating the great biocompatibility of the robot biomaterials. We next studied in vivo biocompatibility of the robot materials by subcutaneously implanting the materials into immunocompetent C57BL/6 mice and analyzing the associated immune responses. C57BL/6 mice were subcutaneously implanted with PDMS containing 25 vol% NdFeB magnetic particles or pure PDMS, followed by the analysis of immune cells at the implantation site after 8 days (Figure 3B,C). No sign of weight loss was observed (Figure 3D). As expected, a small number of CD45^+^ immune cells were detected at the implantation site (Figure 3F), most of which were neutrophils and macrophages that are known to first respond to external materials. Compared to pure PDMS, our robotic materials (PDMS with magnetic particles) showed negligible changes in the number of immune cells including CD11b^+^CD11c^+^ dendritic cells (Figure 3G), CD11b^+^F4/80^+^ macrophages (Figure 3H), and CD11b^+^Gr1^+^ neutrophils (Figure 3I). These data demonstrated the negligible immunogenicity of the robot materials, and the feasibility of implanting a small size of magnetic robot for future mechanotherapy applications (refer to Experimental Section for detailed information.)

### 2.2 Wireless Magnetic Robots with Biaxial Motion

**Figure 4:**
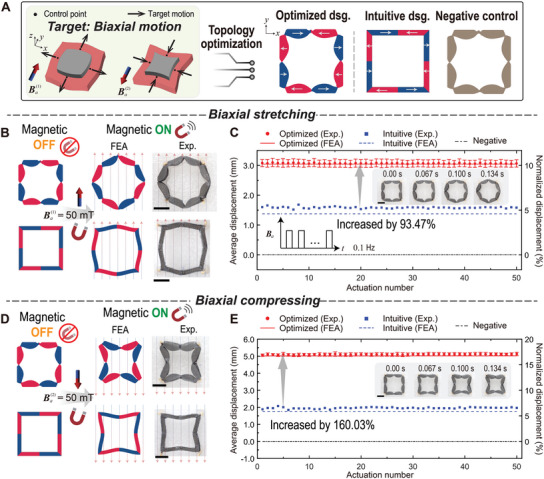
Mechanical performance of the soft robots programmed with biaxial motion. A) Illustrations of the target biaxial motions, the optimized design, intuitive design, and negative control. The color of the members, along with the white arrows, indicates the embedded magnetization directions. B) Biaxial stretching actuation (finite element analysis (FEA) and experiment) of the optimized and intuitive designs under a positive *y*‐direction magnetic field $|B_{a}^{\left(1\right)}|$ = 50 mT. C) Cyclic performance test of the biaxial stretching under the actuation rate of 0.1 Hz (Optimized dsg.: *n* = 3; Intuitive dsg.: *n* = 1). The subplots illustrate the deformation process transitioning from magnetic OFF to magnetic ON. D) Biaxial compressing actuation (FEA and experiment) of the optimized and intuitive designs under a negative *y*‐direction magnetic field $|B_{a}^{\left(2\right)}|$ = 50 mT. E) Cyclic performance test of the biaxial compressing under the actuation rate of 0.1 Hz (Optimized dsg.: *n* = 3; Intuitive dsg.: *n* = 1). The subplots illustrate the deformation process transitioning from magnetic OFF to magnetic ON. See Movie S1 (Supporting Information) for the mechanical performance tests of the soft robots programmed with biaxial motion. Scale bar: 10 mm.

To validate the effectiveness of the optimized robots in providing target mechanical stimulation, we first conducted performance tests on the wireless robots alone, without involving any tissue. **Figure** **4** and Movie S1 (Supporting Information) show the magneto‐mechanical performance tests of robots programmed with target biaxial motions. Figure 4A depicts the target tissue motions: biaxial stretching and compressing under two uniformly distributed external magnetic fields $B_{a}^{\left(1\right)}$ and $B_{a}^{\left(2\right)}$, respectively, with opposite directions along the positive and negative *y*‐axis, each having a magnitude of 50 mT. Taking into account the target tissue motion and mechanical properties of tissues and robots, the topology optimization approach produces one optimized design achieving the target biaxial motions, as depicted in Figure 4A (refer to Section S2, Supporting Information for detailed information on the topology optimization). To demonstrate the actuation performance of the optimized robots, we include a comparison case where the topology and magnetization directions are intuitively designed (see Figure 4A, Intuitive dsg.). Additionally, robots that are non‐magnetized (same topology as the optimized design) are also used as negative controls.

Figure 4B illustrates the undeformed and actuated states of both the optimized and intuitive designs. The results indicate that both the optimized and intuitive designs successfully achieve the target biaxial stretching motions under magnetic actuation qualitatively but with different magnitudes of the actuation displacement. To evaluate the reproducibility of the optimized robots' performance, we conduct cyclic actuation on the three designs at a frequency of 0.1 Hz, with each magnetic on or off state lasting for 5 s. To quantitively assess the actuation performance, we evaluate the average displacement by computing the mean of the actuated displacements at the control points. Additionally, for a more universally applicable representation of displacement, regardless of the robot's dimensions, we introduce the normalized displacement obtained by dividing the average displacement by the distance between the control point to the center of the robot. Figure 4C plots both the numerically predicted and experimentally measured average and normalized displacements over 50 cycles of actuation, which demonstrates the excellent match between the actual displacements and simulation predictions over numerous rounds of actuation. In contrast, the negative control could not be actuated when exposed to the magnetic field due to the lack of remanent magnetization. Notably, the optimization approach is able to increase the actuation performance of the biaxial stretching robots by 93.47%, in comparison with the intuitive design. Similarly, for the biaxial compressing robot, our optimized design is able to achieve the desired motion in the presence of magnetic field ($B_{a}^{\left(2\right)}$) and can increase the actuation performance by 160.03% compared to the intuitive design in a cyclic actuation test (Figure 4D,E). The subplots in Figure 4C,E illustrate the deformation process as it shifts from the magnetic OFF state to the magnetic ON state. It is evident that the robot undergoes rapid deformation under magnetic actuation, achieving this transformation in ≈0.134 s.

### 2.3 Wireless Magnetic Robots with Uniaxial, Shearing, and Dual‐Mode Motions

**Figure 5:**
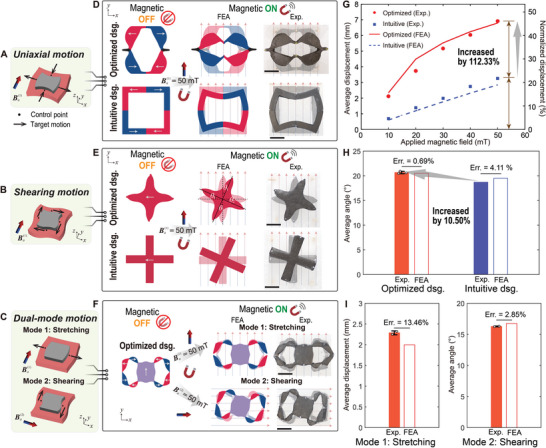
Mechanical performance of the soft robots programmed with uniaxial, shearing, and dual‐mode motions. A–C) Illustration of the target uniaxial, shearing, and dual‐mode (mode 1: stretching; mode 2: shearing) motions. D,E) Actuation (FEA and experiment) of the optimized and intuitive designs under a positive *y*‐direction magnetic field $|B_{a}^{\left(1\right)}|$ = 50 mT for the uniaxial and shearing motions. F) Actuation (FEA and experiment) of the dual‐mode optimized design under positive *y*‐direction and *x*‐direction magnetic fields with $|B_{a}^{\left(1\right)}\left|=\right|B_{a}^{\left(2\right)}|$ = 50 mT, respectively. G) Actuated displacements (FEA and experiment) of the optimized (*n* = 1) and intuitive (*n* = 1) designs programmed with the uniaxial motion under different magnitudes of $B_{a}^{\left(1\right)}$. H) Rotational angles (FEA and experiment) of the optimized (*n* = 3) and intuitive (*n* = 1) designs programmed with the shearing motion under $|B_{a}^{\left(1\right)}|$ = 50 mT. The average rotational angle is calculated by taking the average among the angles θ_*i*_, *i* = 1,.., 4 for each member of the designs. I) Actuated displacements and rotational angles (FEA and experiment) for the optimized design programmed with the dual‐mode motion (*n* = 2). See Movies S2–S4 (Supporting Information) for the mechanical performance tests of the soft robots programmed with uniaxial, shearing, and dual‐mode motions, respectively. Scale bar: 10 mm.

**Figure** **5**A–C illustrates other principal target tissue motions: uniaxial, shearing, and dual‐mode motions. For the uniaxial motion, the robot is designed to stretch in the *x*‐direction and compress in the *y*‐direction. For the shearing motion, the robot is designed to generate clockwise rotation. The dual‐mode design refers to a more challenging case where stretching and shearing motions can be achieved through a single design under different magnetic fields. The optimized designs for these three cases are shown in Figure 5D–F.

In the uniaxial motion case (Figure 5D,G and Movie S2, Supporting Information), the optimized design possesses four members with two magnetization directions. Note that this design also incorporates non‐magnetized regions. The motion at the control points is induced by the magnetic torque generated by the four magnetized members. Inspired by the actuation mechanism in the optimized design, we propose an intuitive design (Figure 5D) for comparison. The simulation and experimental results indicate the target motions can be successfully achieved. We then evaluate the actuation performance by plotting the average and normalized displacement over the control points under varying magnitudes of $|B_{a}^{\left(1\right)}|=10,20,\text{…},50$ mT (Figure 5G). The results exhibit a consistent agreement between the simulation and experimental data. The actuation performance is increased with the increase of the applied magnetic field. Furthermore, the optimized design consistently outperforms the intuitive design across varying magnetic fields. It's important to note that this relationship holds true for other actuation modes as well.

In Figure 5E,H and Movie S3 (Supporting Information), we present the performance test of the robot programmed with the shearing motion. In this case, a cross‐shaped topology connecting the four control points with uniform magnetization is generated by the optimization framework. Under the actuation of the external magnetic field, the robot rotates to align its magnetization with the applied magnetic field, resulting in shearing among the control points. Similarly, we incorporate an intuitively designed robot for comparison. The numerical and experimental demonstration figures for the undeformed and deformed states under $|B_{a}^{\left(1\right)}|=50$ mT are shown in Figure 5E. We then evaluate the average rotational angle and plot the comparison results in Figure 5H, indicating a high agreement between the numerical predictions and experimental results, with an error of 0.69% and 4.11%. In this particular target motion setup, the optimized design exhibits only a relatively moderate improvement in actuation performance compared to the intuitive design, with an increase of 10.50%. This is due to the fact that the intuitive design proposed for this specific case is close to the optimized solution.

When considering the dual‐mode motion (Figure 5F,I and Movie S4, Supporting Information), achieving precise and simultaneous control of two different motions with a single design based solely on intuition poses significant challenges. However, by leveraging our topology optimization framework, we successfully obtain the design depicted in Figure 5F. Notably, the stretching motion primarily originates from the movement generated by the eight surrounding members positioned on the left and right sides adjacent to the central part of the design. Conversely, the shearing motion is predominantly facilitated by the rotation of the central part of the design. To assess the performance of the design, we compare the average displacement at the two control points for the stretching mode and the average rotating angle for the shearing mode, as illustrated in Figure 5I. The comparison between the numerical and experimental results reveals an error of 13.46% and 2.85% for the stretching and shearing modes, respectively.

The aforementioned performance tests validate that, when provided with a target tissue motion, our proposed design framework has the ability to automatically generate biomaterials exhibiting superior actuation performance compared to designs obtained solely from intuitive approaches. The experimental validation confirms the replicability of the fabricated designs. The establishment of this design framework holds great promise for the creation of mechanotherapy soft robots capable of accommodating diverse modes of loading, taking into account the unique properties of individual tissues.

### 2.4 Wireless Magnetic Robots‐Induced Biaxial Motion of Different Tissues

**Figure 6:**
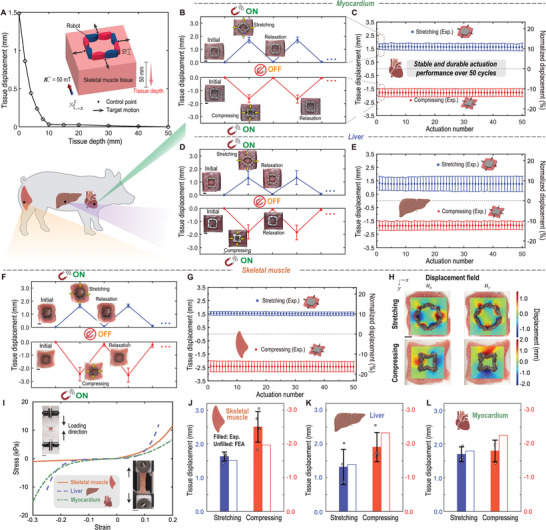
Ex vivo application of the soft robots programmed with biaxial stretching/compressing motions on different types of tissues. A) FEA result of the correlation between actuated tissue displacement and tissue depth for the robot with biaxial stretching motion. The inset shows the schematic of the simulation setup. B,C) Experimental measurements of average displacements at control points within myocardial tissue (*n* = 3): B) over two cycles of magnetic ON and OFF, and C) across 50 actuation cycles. D,E) Experimental measurements of average displacements at control points within liver tissue (*n* = 4): D) over two cycles of magnetic ON and OFF, and E) across 50 actuation cycles. F,G) Experimental measurements of average displacements at control points within skeletal muscle tissue (*n* = 5): F) over two cycles of magnetic ON and OFF, and G) across 50 actuation cycles. H) Displacement fields (left column: *u*_*x*_; right column: *u*_*y*_) acquired by the DIC analysis of the skeletal muscle tissue deformation actuated by the biaxial stretching (top row) and compressing (bottom row) motions of the optimized robot. I) Stress–strain relationships of skeletal muscle, liver, and myocardium tissues. Subplots demonstrate the uniaxial tension and compression tests. J–L) Comparison of experimentally measured and numerically predicted average stretching and compressing displacements at control points in skeletal muscle tissue (*n* = 5), liver tissue (*n* = 4), and myocardium tissue (*n* = 3) during one‐time actuation. The experimental data are presented as mean ± SD. The magnitude of the applied magnetic fields is 50 mT. See Movies S1 and S5 (Supporting Information) for the ex vivo application of the soft robots programmed with biaxial motion. Scale bar: 10 mm. The pig outline in (A) was created with BioRender.com.

To demonstrate the capability of the optimized robots in generating and delivering mechanical stimulation to the tissue, we conduct ex vivo testing of the biaxial robots on different types of tissues, including porcine skeletal muscle, liver, and myocardium tissues (**Figure** **6**).

Initially, a three‐dimensional (3D) numerical simulation is conducted to explore the penetration depth of deformation propagating into the tissue bulk (Figure 6A). Specifically, we consider the biaxial stretching robot implanted on a bulky porcine skeletal muscle tissue with a depth of 50 mm. Subsequently, we calculate tissue displacement as the mean of actuated displacements across four control points at different depths within the tissue bulk, employing a magnetic actuation of 50 mT. It can be seen despite a reduction in tissue deformation with increasing depth, displacement remains observable within the 0‐10 mm depth range. For the following ex vivo tests, tissue samples with an approximate depth of 5–10 mm are adopted. We attach optimized robots to the surface of tissues via the super glue. While super glue is used as an adhesive between soft robots and tissues in this study for demonstration purposes, we will explore the use of different types of bioadhesives such as COSEAL, cyanoacrylates, and tough hydrogel adhesives to ensure a high adhesion strength and good biocompatibility. These bioadhesives have been widely used for in vivo hemostatic, tissue repair, and imaging applications.^[^ ^38^, ^39^, ^40^, ^41^, ^42^, ^43^, ^44^ ^]^ To enable the real‐time monitoring of displacement via digital image correlation (DIC), we spray speckle patterns onto the surface of tissues (Figure S4, Supporting Information). Under the actuation of the magnetic field, the robot's movement leads to the deformation of the underlying tissue.

To assess the stability of the actuation performance in stimulating tissue deformation, we employ a cyclic loading strategy for the ex vivo test at an actuation rate of 0.1 Hz,^[^ ^10^ ^]^ ensuring sufficient deformation and recovery processes for the tissue and robot. Experimental measurements of average displacements at control points in three types of tissues (myocardium, liver, and skeletal muscle) during two cycles of magnetic activation and deactivation for biaxial stretching and compressing are presented in Figure 6B,D,F, respectively. Insets accompanying the figure display photos of tissues during the actuation. Note that slight residual displacements are observed in the relaxing state when the magnetic field is off, attributed to the viscoelasticity of the bio‐tissues. In Figure 6C,E,G, the average tissue displacements and their corresponding normalized values over 50 consecutive actuation cycles are illustrated. These normalized displacements are computed by dividing the average tissue displacements by the distance between the control points and the center of the robot. It can be seen that for all three types of tissues, the actuation performance can be sufficiently maintained after 50 cycles of actuation. Figure 6H illustrates the experimentally measured displacement field of skeletal muscle tissue during biaxial stretching and compressing motions, acquired through DIC analysis. This presentation further confirms the successful attainment of the target tissue deformations.

To capture the hierarchical interaction between the biological tissue and robots and predict tissue deformation, we characterize and model the nonlinear mechanical properties of the tissues. From the experimentally measured stress‐strain curves (Figure 6I), it can be seen the tissues exhibit a significant degree of nonlinearity. Given the inherent large variability in biological tissue properties, ensuring data reliability is crucial. In this context, it is demonstrated that the measured data falls within a reasonable range when compared to values reported in the literature.^[^ ^45^, ^46^, ^47^, ^48^, ^49^, ^50^ ^]^ In this work, we employ a hyperelastic Ogden model^[^ ^51^ ^]^ to characterize the nonlinear mechanical properties of these three types of tissues (refer to Section S3 and Figure S1, Supporting Information, for detailed information on the tissue characterization). With the characterized material property of the tissue and robot, we can conduct rational FEA on the tissue's nonlinear mechanical responses under the actuation of the robot and perform experimental validation. To evaluate the accuracy of the optimization and numerical modeling approach in capturing the interaction between the tissues and the robots, we quantitatively compare the average displacements at the control points under 1‐time actuation for these three types of tissues shown in Figure 6J–L. It can be seen the FEA results lie in the reasonable range of the experimental results. For the biaxial stretching case, we observe comparable deformations in skeletal muscle and myocardium tissues, while the liver tissue displays unexpectedly smallest actuation deformation. This observation is rationalized by our characterization data (Figure 6I), which reveals that despite its lower initial modulus, the liver undergoes premature stiffening relative to the other tissues. Consequently, it exhibits minimal actuation displacement under the regime of large deformation. Regarding biaxial compression, the liver and myocardium tissues exhibit comparable deformation levels, whereas the skeletal muscle tissue displays the largest experimentally measured actuation displacement. This is attributed to the skeletal muscle's lower stiffness under compressive strain (Figure 6I). It is noteworthy that the significant biaxial compressive deformation in skeletal muscle tissue is not adequately predicted by FEA due to limitations in the adopted model to capture tension‐compression asymmetry in skeletal muscle. Addressing this limitation requires the utilization of more refined models in future investigations.

The above findings provide compelling evidence of the potential of the optimized wireless robots to proficiently transmit mechanical stimuli and induce biaxial stretching and compressing deformations of diverse biological tissues. This indicates a promising direction for future research and development, leveraging the design framework to create mechanotherapy soft robots that are specifically tailored to the unique properties and requirements of individual tissues. By incorporating tissue‐specific considerations into the design process, these robots hold the prospect of unlocking new avenues for targeted therapeutic interventions, optimizing the delivery of mechanical stimulation, and maximizing the therapeutic benefits across a wide range of tissue types.

### 2.5 Wireless Magnetic Robots‐Induced Uniaxial Motion of Skeletal Muscles

**Figure 7:**
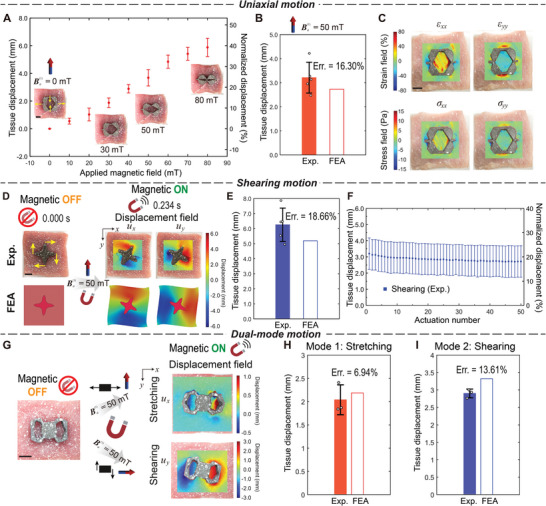
Ex vivo application of the soft robots programmed with different target motions on skeletal muscle tissue. A) Average and normalized tissue displacements at the control points evaluated under different magnitudes of the applied magnetic field $B_{a}^{\left(1\right)}=0,10,20,\text{…},80$ mT for robots programmed with the uniaxial motion (*n* = 2). Insets show photos of the tissue during actuation. B) Comparison of experimentally measured and numerically predicted average uniaxial motion at control points under $B_{a}^{\left(1\right)}=50$ mT during one‐time actuation (*n* = 5). C) Strain (top) and stress (bottom) fields of the skeletal muscle tissue actuated by the uniaxial motion of the optimized robot (plotted on the undeformed configuration). D) Displacement fields (left: *u*_*x*_; right: *u*_*y*_) acquired by the DIC analysis (top) and FEA (bottom) of the skeletal muscle tissue actuated by the shearing motion of the optimized robot. E) Comparison of experimentally measured (*n* = 5) and numerically predicted average shearing motion at control points during one‐time actuation. F) Experimentally measured average and normalized shearing motion for 50 times of actuation (*n* = 5). G) Displacement fields acquired by the DIC analysis of the skeletal muscle tissue actuated by the dual‐mode motion of the optimized robot: *u*_*x*_ of the stretching mode (top); *u*_*y*_ of the shearing mode (bottom). H,I) Comparison of experimentally measured (*n* = 3) and numerically predicted average dual‐mode motion at control points during one‐time actuation: H) mode‐1 stretching and I) mode‐2 shearing. The experimental data are presented as mean ± SD. See Movies S2, S3, and S4 (Supporting Information) for the ex vivo application of the soft robots programmed with uniaxial, shearing, and dual‐mode motions, respectively. Scale bar: 10 mm.

Skeletal muscle has been an active target of research for mechanical stimulation and mechanotherapy.^[^ ^6^, ^10^ ^]^ In addition to biaxial motion, we next evaluate the capability of the wireless magnetic robot to induce the uniaxial motion of underlying porcine skeletal muscles (**Figure** **7**A–C and Movie S2, Supporting Information). As expected, the uniaxial wireless robots are able to stretch the porcine skeletal muscle tissue in the horizontal direction while compressing the tissue in the vertical direction. The average and normalized tissue displacements at four control points increases with the magnitude of the magnetic field ($|B_{a}^{\left(1\right)}|=0,10,20,\text{…},80$ mT) (Figure 7A), demonstrating that varying the external applied magnetic field allows for control of different levels of actuation. Figure 7B compares the experimental (with *n* = 5 independent samples) and FEA results under $|B_{a}^{\left(1\right)}|=50$ mT. Figure 7C depicts the experimentally measured strain fields acquired through the DIC analysis for skeletal muscle tissue deformation. We can see that under the programmed uniaxial motion of the robot, the inner middle part of the tissue sample is stretched in the horizontal direction while being compressed in the vertical direction. Conversely, the outer small regions surrounding the left and right control points undergo compression in the horizontal direction, while the outer regions surrounding the top and bottom control points undergo stretching in the vertical direction, driven by the robot's movement. To evaluate the generated stress on the tissue sample, we numerically calculate the stress field based on the characterized stress–strain relationship (Figure 6I) of the skeletal muscle tissue as shown in Figure 7C.

### 2.6 Wireless Magnetic Robots‐Induced Shearing Motion of Skeletal Muscles

We next studied whether the wireless robots optimized for a shearing motion can induce the designed mechanical stimulation of skeletal muscles (Figure 7D–F and Movie S3, Supporting Information). In the presence of a magnetic field (50 mT), the optimized robot, together with the underlying skeletal muscle tissue, shows the expected clockwise rotation (Figure 7D). In Figure 7D, we plot the experimental and numerical displacement fields during the actuation. It can be seen the top and bottom control points of the tissue experience horizontal shearing motion while the left and right control points experience vertical shearing motion satisfying the design target. The FEA results can successfully predict the deformation trend of the skeletal muscle tissue. Figure 7E plots the average shearing displacement comparison at the control points between the numerical and experimental results for the 1‐cycle actuation. We further evaluate the sustainability of the shearing motion robots by applying numerous cyclic actuations. The average and normalized shearing displacements over the last 50 cycles of actuation are plotted in Figure 7F for reference. The results indicate that the observed displacements in the subsequent cycles exhibit a reduction compared to those in the initial cycle. This decline can be attributed to the inherent viscoelastic properties of the tissue. Nonetheless, even after multiple cycles of loadings, a substantial tissue displacement of ≈3 mm (20% normalized displacement) can still be achieved.

### 2.7 Wireless Magnetic Robots‐Induced Multimodal Motion of Skeletal Muscles

Figure 7G‐I and Movie S4 (Supporting Information) show the experimental results of the multi‐modal motion robots on the skeletal muscle tissue. In the stretching mode, where only horizontal deformation is controlled as the target, we display the corresponding *u*_*x*_ displacement field in the top figure of Figure 7G. Similarly, in the shearing mode, where only vertical deformation is controlled, we show the *u*_*y*_ displacement field in the bottom figure of Figure 7G. It can be observed under the vertical magnetic field $B_{a}^{\left(1\right)}$ (50 mT), the robot can induce the stretching movement of the underlying tissue surrounding the left and right control points. The average tissue displacement is measured to be 2.04 mm, resulting in an error of 6.94% compared to the predicted value (Figure 7H). When subjected to the horizontal magnetic field $B_{a}^{\left(2\right)}$ instead, the robot is capable of generating the shearing motion of the tissue at the two control points, with an average displacement of 2.90 mm. This induces an error of 13.61% compared to the numerical result (Figure 7I). These experiments demonstrate that the optimized robots programmed with different target motions can successfully transfer the mechanical stimulation to the underlying tissues and induce the deformation of tissues in a predictable manner.

## 3 Discussion

Conventional physical therapy or commercialized physiotherapy is commonly used for muscle mechanotherapy in humans. There are some scenarios where miniature remote‐controllable mechanotherapy would be preferred, including 1) companion animals (e.g., cats and dogs) with relatively smaller muscle sizes and 2) internal muscles such as cardiac muscles. In this work, we developed wireless magneto‐active soft robots that can be remotely actuated by magnetic fields to exhibit various types of motions (i.e., biaxial stretching and compressing, uniaxial motion, shearing, and multimodal motions) and induce the deformation of different tissues in a precisely programmable and predictable manner, providing feasibility for future clinical translation.

We first employ an inverse design paradigm to create a set of topology‐optimized wireless magneto‐active soft robots, each tailored for specific principal actuation modes. Then, we establish a biocompatible fabrication protocol using a mold‐casting approach. Through this approach, the robots can be manufactured at various scales while ensuring reproducibility. These soft robots show good biocompatibility through in vitro and in vivo tests on mice. Finally, we conduct the ex vivo performance test on different types of porcine tissues to validate the effectiveness and precision of the fabricated robot designs. The experimental results confirm that the magneto‐active robots are capable of transferring controlled motion to the underlying tissue. The numerical results yield reasonable predictions on the deformations of different types of tissues under various actuation modes by considering the characterized properties of robot and tissue materials. We observe a good agreement between the FEA and experimental results in general. Quantitative mismatch observed in some cases can be attributed to the high variations in tissue properties, manufacturing imperfection, and the limitations of the adopted model for tissues, such as the neglect of the anisotropy of tissues, which can be further improved by adopting more refined models.

Overall, this study highlights the effectiveness of magneto‐active robots in stimulating various modes of deformations in bio‐tissues in a controllable and programmable manner, which has great potential for facilitating both fundamental mechanotransduction studies and the development of new‐generation mechanotherapy for tissue repair. In an effort to move towards the potential clinical translation of our magneto‐active robots, we will test their efficacy for muscle restoration in a mouse model of ischemic muscle injury, as well as the associated immune response and potential acute and chronic side effects. Further, we will test the performance of the magneto‐active materials for muscle repair in larger animal models such as rats and pigs. With further improvement of our magneto‐active robots based on the lessons we learn from the in vivo tests, we will also explore their use for the repair of internal tissues such as heart, liver, kidney, and cartilage.

For the future muscle restoration study in a mouse model, while increasing evidence indicated the promise of cyclic mechanical loading to regulate local immune responses and facilitate tissue restoration,^[^ ^6^, ^10^ ^]^ the optimal mechanical force, tissue deformation, and cycles of loading remain unknown. These parameters will be taken into consideration for the design of animal studies. In cases when the deformation of the robot and underlying tissue could be limited due to the presence of friction between tissue layers, the mechanical forces can be amplified by adjusting the percentages of magnetic particles in the soft robot and/or the external magnetic field. Another challenge lies in visualizing the in vivo deformation of hard magnetic soft robots. Imaging modalities could be explored as potential solutions to address this issue. While superglue was used in the in vitro and ex vivo tests in this work, clinically relevant bioadhesives including Dermabond, DuraSeal, Tisseel, BioGlue, Coseal, and Histoacryl will be explored for in vivo studies. These bioadhesives can stably attach to the underlying wet tissues with a decent adhesion strength (up to 20 J m^−2^ interfacial toughness, 50 kPa shear strength, and up to 50 kPa tensile strength for skin tissues).^[^ ^40^, ^52^ ^]^ We envision that our magneto‐active robots can be stably attached to the tissue via Dermabond or other clinically relevant bioadhesives for days to weeks, and can be easily removed via a mini‐surgery after use.

To conclude, this work represents our first effort in the design and validation of a new programmable magneto‐active robot for potential mechanotherapy applications. While there is still a long way to go, the magneto‐active robots with excellent biocompatibility, scalability, and ability to apply programmable strengths and durations of mechanical force to tissues hold great promise for eventual clinical translation.

## 4 Experimental Section

### Materials and Instrumentation

PDMS (Dow Sylgard 184 Kit), Fetal Bovine Serum (FBS), Calcein AM, and Ethidium Homodimer‐1 were purchased from Thermofisher (Waltham, MA, USA). NdFeB microparticles were purchased from Magnequench (Indianapolis, IN, USA). The magnetic particles were in the form of powder with irregular shapes. The particle size distribution was as follows: D10 < 13 microns, D50 is 20‐30 microns, and D90 < 55 microns (MQFP‐B+‐20441‐088). Figure S3 (Supporting Information) shows two representative optical microscopy images of the microstructure of the hard magnetic soft material filled with 25 vol% NdFeB microparticles at two different magnifications, captured by a Keyence VK‐X1000 3D laser scanning confocal microscope. PVA was purchased from Prusa Research (Prague, Czech Republic). Sil‐poxy adhesive and Ecoflex 00‐30 were purchased from Smooth‐On Inc. (Macungie, PA, USA). Porcine tissues were purchased from Sierra for Medical Science (Whittier, CA, USA). The magnetizer (IM‐10‐30) was purchased from ASC Scientific (Narragansett, RI, USA). Helmholtz coils were purchased from Woodruff Scientific (Santa Fe, NM, USA). Photos and videos of materials were taken with a SONY α7R camera. Deformations of robots and tissues were tracked via DIC and Tracker. Fluorescence‐activated cell sorting (FACS) analyses were collected on Attune NxT flow cytometers and analyzed on FCS Express v6 and v7. Mechanical tests of robots and tissues were performed on the Instron 68TM‐30.

### Cell Line and Animals

3T3‐L1 cell line was purchased from American Type Culture Collection (Manassas, VA, USA). Cells were cultured in RPMI 1640 containing 10% FBS, and 100 units/mL Penicillin/streptomycin at 37°C in 5% CO_2_ humidified air. Female C57BL/6 mice were purchased from Jackson Laboratory (Bar Harbor, ME, USA). Feed and water were available ad libitum. Artificial light was provided in a 12/12 h cycle. All procedures involving animals were done in compliance with National Institutes of Health and Institutional guidelines with approval from the Institutional Animal Care and Use Committee at the University of Illinois at Urbana‐Champaign, IACUC #23037.

### Fabrication of Robots

PDMS and NdFeB particles were mixed thoroughly for 15 min, followed by defoaming for 1 h to eliminate any trapped air bubbles. For the curing process, PVA molds with various geometries were 3D printed and used. Each geometry corresponds to an individual component of the optimized design. The mixture was then cured at 80°C for 2 h. After curing and demolding, the individual components were magnetized according to the desired magnetization directions using a 2 T impulse magnetic field (IM‐10‐30), as recommended by the manufacturer's datasheet, ensuring it is deemed sufficient to attain ≥95% magnetic saturation of the NdFeB particles. Subsequently, different parts were bonded together in a complete PVA mold using Sil‐poxy adhesive. Once the adhesive was fully cured at room temperature, the integrated robots were removed from the complete mold. The size of the robot can be simply scaled to any dimension. In this study, a size of 30 mm (length) × 30 mm (width) × 10 mm (thickness) design domain of the robot was used.

### Magnetic Actuation System

A pair of Helmholtz coils with the radius and spacing of 50 mm was utilized to generate a nearly uniform magnetic field in alignment with the computational assumption. The direction and magnitude of the generated magnetic field were measured by a Gauss meter (PCE‐MFM 4000). The Helmholtz coil was connected to a programmable power supply. The magnitude of the generated magnetic field was controlled to be consistent with the simulation parameters by adjusting the supplied current. The measurement setup of the magnetic field is shown in Figure S5 (Supporting Information). With respect to the center of the reference system defined in Figure S5 (Supporting Information), the robot experiments were conducted within a 30 mm‐wide workspace along the *x* and *y* axes. Prior to each experiment, precise calibration of the robot's location was performed to ensure it remained within the space of uniform and consistent magnetic fields.

### Performance Test of Robots

To conduct the performance test of the manufactured robots, first, the bottom surface of the robot was bonded to a thin connector (see Figure S4, Supporting Information) made of Ecoflex 00‐30 using the Sil‐poxy adhesive. The experiment setup for testing the performance of robots is illustrated in Figure S4 (Supporting Information). A pair of Helmholtz coils was used to generate a nearly uniform magnetic field. The robot was placed at the center of the coils. To fix the robot, slender bars were inserted into the bottom connector at its four corners and affixed to a foam support. The robot was elevated slightly creating a small gap to prevent friction during its movement under actuation. A camera (SONY α7R) was positioned appropriately to record the videos. The displacements of the control points on the robots were then obtained by postprocessing the video using Matlab and Tracker.

### Actuation of Porcine Tissues

For the ex vivo actuation and deformation experiments on porcine liver, myocardium, and muscle tissues (see Figure S4, Supporting Information), the tissue samples were prepared in a nearly rectangular shape. The robot was bonded on the top surface of the tissue by super glue. The tissue surface was delicately dried first, and then the super glue (Loctite 1365882, Amazon) was applied to the bottom of the soft robots. Subsequently, the robots were applied to the tissues, and any surplus glue residue was removed from the tissue. To ensure secure adhesion, gentle pressure was applied for a duration of 2 min. The displacements of the tissue surrounding the control points were video recorded and tracked by Tracker. To analyze the full displacement field of the tissue, speckle patterns were sprayed onto the surface of the tissue. The recorded videos were then postprocessed by Matlab and analyzed using Ncorr,^[^ ^53^ ^]^ a two‐dimensional (2D) DIC program. The displacement fields were created by comparing the images of the tissue samples before and after the actuation. The areas covered by the robots were excluded from the analysis. Note that since the magnetic actuation was quite fast and the induced tissue strain was relatively larger, intermediate actuation images were added and the high strain and backward analysis were enabled to make the DIC work effectively. Further details are provided in Section S3.3 (Supporting Information).

### Mechanical Characterization of Robotic Materials

The mechanical properties of the robotic materials were characterized by fitting the parameters in the constitutive model (Section S1, Supporting Information) to the stress–strain relationships obtained from testing dog‐bone specimens according to standard^[^ ^54^ ^]^ in uniaxial tension, and cylinder specimens according to standard^[^ ^55^ ^]^ in compression (Figure S2, Supporting Information). For each group of materials (20:1 PDMS elastomer with 0 vol%, 15 vol%, and 25 vol% NdFeB magnetic particles), three samples for tension or compression were tested on a loading machine (Instron 68TM‐30) with a 5.0 mm min^−1^ overhead speed. To accurately simulate the behavior in the performance test (Figures 4 and 5), the mechanical property of the Ecoflex connector was also tested and characterized using the *I*_1_‐based hyperelastic model by performing the uniaxial tension test using dogbone samples. The residual magnetic flux densities of the material were measured using a vibrating‐sample magnetometer (Quantum Design MPMS3).

### In Vitro Biocompatibility Test

The biocompatibility of the robotic materials was assessed in seven different groups: 1) In‐plane magnetized 25 vol% NdFeB PDMS (*n* = 6), 2) Out‐of‐plane magnetized 25 vol% NdFeB PDMS (*n* = 6), 3) Non‐magnetized 25 vol% NdFeB PDMS (*n* = 6), 4) In‐plane magnetized 15 vol% NdFeB PDMS (*n* = 6), 5) Out‐of‐plane magnetized 15 vol% NdFeB PDMS (*n* = 6), 6) Non‐magnetized 15 vol% NdFeB PDMS (*n* = 6), and 7) pure PDMS (n = 6). Circular disks of materials with a diameter of 10 mm and a thickness of 3 mm were placed in 24‐well plates, subjected to UV sterilization for 1 h, and washed with phosphate‐buffered saline (PBS) prior to use. 3T3‐L1 cells from American Type Culture Collection (Manassas, VA, USA) were then placed on top of the materials and cultured in Dulbecco's modified Eagle's medium (DMEM) containing 10% FBS, 100 units mL^−1^ Penicillin G and 100 µg mL^−1^ streptomycin at 37 °C for 48 h in 5% CO_2_. To assess cell viability, live and dead cells were stained with Calcein AM and Ethidium Homodimer‐1, respectively, and analyzed on a flow cytometer.

### In Vivo Biocompatibility Test

C57BL/6 mice were divided into three groups: 1) In‐plane magnetized 25 vol% NdFeB PDMS (*n* = 3), 2) pure PDMS (*n* = 3), and 3) No treatment (*n* = 3). On day 0, a small incision was cut on the back skin of immunocompetent C57BL/6 mice. Materials were then placed in the subcutaneous pocket, followed by suture closing. The body weight of mice was closely monitored after the implantation. On day 8, tissues surrounding the implants were harvested, and treated with collagenase IV (0.5 mg mL^−1^) for 45 min. Following the collagenase IV treatment, tissues were disrupted using a syringe plunger to release cells. These released cells were then collected, washed, and subjected to staining for flow cytometry analysis. For the analysis of immune cell populations, cells were stained with APC‐conjugated anti‐CD45, PE‐conjugated anti‐CD11b, PE/Cy7‐conjugated anti‐CD11c, Alexa Fluor 700‐conjugated anti‐Ly‐6G/Ly‐6C, PerCP/Cy5.5‐conjugated anti‐F4/80.

### Mechanical Characterization of Porcine Tissues

For the characterization of porcine biceps femoris muscle tissue, liver, and myocardium tissues, the tissue samples were cut into strips and cubics for the uniaxial tension and compression tests, respectively. The tests were performed at room temperature using a loading machine (Instron 68TM‐30) at a strain rate of 0.5% s^−1^. Detailed information is provided in Section S3 and Figure S1 (Supporting Information).

### Topology Optimization Approach

Utilizing a 2D topology optimization framework tailored for magnetic‐actuated materials, the in‐plane geometry and remnant magnetization distribution of biomaterial robots within a tissue environment were optimized. The externally applied magnetic field was considered to be a uniform vector, with a magnitude of 50 mT. To parameterize the entire design, two sets of design variables were introduced. The matrix material distribution, representing geometry, was parameterized by the physical density variable $\bar{\rho }$. Here, ${\bar{\rho }}_{e}=1$ designates a solid element *e*, while ${\bar{\rho }}_{e}=0$ designates a void. Concurrently, a set of magnetization indicator variable vectors ${\bar{m}}_{e}^{\left(j\right)},j=1,\text{…},N_{m}$ is employed to express the actual residual magnetic flux density $B_{r,e}$. Formally, the residual magnetic flux density in element *e* is defined as

(1) $$B_{r,e}=\sum_{j=1}^{N_{m}}{\left({\bar{m}}_{e}^{\left(j\right)}\right)}^{p_{m}}B_{r}^{\left(j\right)},$$

where ${\bar{m}}_{e}^{\left(j\right)}=1$ indicates the selection of the *j*th candidate residual magnetic flux density $B_{r}^{\left(j\right)}$, while ${\bar{m}}_{e}^{\left(j\right)}=0$ signifies that the *j*th candidate residual magnetic flux density $B_{r}^{\left(j\right)}$ is not chosen. To promote the convergence of the physical magnetization variables ${\bar{m}}_{e}^{\left(j\right)}$ to either 1 or 0, a penalization power was introduced, denoted as *p*_m_.

To describe the nonlinear magneto‐mechanical performance of magnetic robots and tissues, an interpolation of the energy function based on the physical variables $\bar{\rho }$ and ${\bar{m}}^{\left(j\right)}$ was presented, where *j* = 1,…, *N*_*m*_. The expression for the interpolated element‐wise energy $W_{e}^{\left(\ell \right)}$ was provided by the following equation:

(2) $$\begin{array}{c} W_{e}^{\left(\ell \right)}\left({\bar{\rho }}_{e},{\bar{m}}_{e}^{\left(1\right)},\text{…},{\bar{m}}_{e}^{\left(N_{m}\right)},u_{e}^{\left(\ell \right)}\right)=\\ \left[\varepsilon +\left(1-\varepsilon \right){\left({\bar{\rho }}_{e}\right)}^{p_{\rho }}\right]W_{E,e}\left(u_{e}^{\left(\ell \right)}\right)\\ +{\left({\bar{\rho }}_{e}\right)}^{p_{\rho }}W_{M,e}\left(u_{e}^{\left(\ell \right)},B_{r,e}\left({\bar{m}}_{e}^{\left(1\right)},\text{…},{\bar{m}}_{e}^{\left(N_{m}\right)}\right)\right)+W_{T,e}\left(u_{e}^{\left(\ell \right)}\right), \end{array}$$

where $u_{e}^{\left(\ell \right)}$ represents the displacement vector in element *e* under the *l*th applied magnetic field $B_{a}^{\left(\ell \right)}$, and ϵ = 10^−5^ serves as a small value to prevent singular stiffness. The penalization parameters *p*_ρ_, associated with both energies, are employed to penalize both elastic‐stored energy and magnetic free energy, promoting a discrete design.^[^ ^27^ ^]^ The expressions *W*_*E*, *e*_ and *W*_*M*, *e*_ represent the elastic energy and magnetic energy associated with the magnetic robot, respectively. The term *W*_*T*, *e*_ corresponds to the stored energy of underlying tissues, explicitly considered within the optimization framework. For a detailed expression of the energy functions, please refer to Section S1 (Supporting Information).

Having introduced the design space parameterization and free‐energy interpolation schemes, the topology optimization formulation for generating magneto‐active bio‐robots was outlined. The mesh $\Omega_{h}$ consists of *N*_*e*_ elements and *N*_*n*_ nodes. The objective of the topology optimization was to maximize tissue displacements at control points while adhering to constraints and ensuring nested equilibrium. Formally, the topology optimization problem is expressed as follows:

(3) $$\begin{array}{cc} \min_{\begin{array}{c} \rho ,\xi^{\left(1\right)},\text{…},\xi^{\left(N_{m}\right)} \end{array}} & \max_{\begin{array}{c} \ell \in \{1,\text{…},N_{\ell }\}\\ \alpha \in \{1,\text{…},N_{\alpha }^{\left(\ell \right)}\} \end{array}}u_{\alpha }^{\left(\ell \right)},\\ \;\text{s.t.:}\;\; & \frac{v^{T}\bar{\rho }}{|\Omega_{h}|}\leq v_{\max},\\ & \sigma_{0}^{\left(\ell \right)}\leq \sigma_{\max}^{\left(\ell \right)},\;\ell =1,\text{…},N_{\ell },\\ & R\left(\bar{\rho },{\bar{m}}^{\left(1\right)},\text{…},{\bar{m}}^{\left(N_{m}\right)},u^{\left(\ell \right)}\right)=0,\;\ell =1,\text{…},N_{\ell },\\ & 0\leq \rho \leq 1,\\ & 0\leq \xi^{\left(j\right)}\leq 1,\;j=1,\text{…},N_{m}, \end{array}$$

where $u_{\alpha }^{\left(\ell \right)}$ represents the actual displacement at the *α*th control degree of freedom (DOF) of the underlying tissue under the applied magnetic field $B_{a}^{\left(\ell \right)}$. A min‐max formulation^[^ ^56^ ^]^ is employed to maximize the displacement at the control point, with the appropriate sign for the desired deformation modes. The vector v collects element volumes, with its *e*th component *v*_*e*_ denoting the volume of element *e*, and *v*_max_ denotes the prescribed upper bound for volume fraction. To mitigate thin members and restrict excessive local deformations in optimized designs, we incorporate a stress constraint for each applied magnetic field. Here, $\sigma_{0}^{\left(\ell \right)}$ approximates the maximum stress across the design domain, while $\sigma_{\max}^{\left(\ell \right)}$ serves as the upper limit for stress. The equilibrium $R=\frac{\partial \sum_{e}W_{e}^{\left(\ell \right)}}{\partial u^{\left(\ell \right)}}=0$ of the robot‐tissue system (neglecting body force and external mechanical traction) is embedded in the formulation, where the displacement vector $u^{\left(\ell \right)}$ is solved using FEA for each optimization iteration. The gradient‐based optimization problem is addressed using the method of moving asymptotes (MMA).^[^ ^57^ ^]^ For a more comprehensive understanding of the topology optimization framework, refer to Section S2 in the Supporting Information.

### Finite Element Simulation

The evaluation of both optimized robot actuation performance and tissue deformations was conducted utilizing the nonlinear finite element method under large deformation. By adopting a total Lagrangian formulation^[^ ^58^ ^]^ and excluding traction and body force, a displacement‐based finite element problem with the total potential energy is established, expressed as:

(4) $$\Pi \left(u^{\left(\ell \right)}\right)=\sum_{e}\left(W_{E,e}\left(u_{e}^{\left(\ell \right)}\right)+W_{M,e}\left(u_{e}^{\left(\ell \right)}\right)+W_{T,e}\left(u_{e}^{\left(\ell \right)}\right)\right)$$

where *W*_*E*, *e*_ and *W*_*M*, *e*_ represent the elementwise stored energy for the matrix material and magnetic energy for the magnetic robot, respectively, and *W*_*T*, *e*_ denotes the elementwise stored energy for the tissue (or connector). The detailed models adopted are elaborated in Section S1 (Supporting Information).

Minimizing the total potential energy $\Pi (u^{\left(\ell \right)})$ with respect to the global displacement vector u results in the discretized stationary condition:

(5) $$R\left(u^{\left(\ell \right)}\right)=\frac{\partial \Pi }{\partial u^{\left(\ell \right)}}\left(u^{\left(\ell \right)}\right)=F_{\mathrm{int}}\left(u^{\left(\ell \right)}\right)=0$$

which governs the equilibrium of the discretized system. The terms R and **F**_int_ refer to the global residual vector and global internal force vectors, respectively. In this study, the nonlinear equation (5) is solved using the Newton‐Raphson method, with implementation carried out on the Matlab platform. Further details are provided in Section S1 of the Supporting Information.

### Statistical Analyses

Statistical analysis was performed using GraphPad Prism v6 and v8. Sample variance was tested using the F‐test. For samples with equal variance, the significance between the groups was analyzed by a two‐tailed student's t‐test. For samples with unequal variance, a two‐tailed Welch's t‐test was performed. For multiple comparisons, a one‐way analysis of variance (ANOVA) with post hoc Fisher's least significant difference (LSD) test was used. The results were deemed significant at 0.01 < *p ⩽ 0.05, highly significant at 0.001 < **p ⩽ 0.01, and extremely significant at ***p ⩽ 0.001.

## Conflict of Interest

The authors declare no conflict of interest.

## Author Contributions

X.S.Z. supervised the research; X.S.Z. and H.W. conceived the project; C.W. and A.A.S. fabricated the materials; C.W. and J.H. performed experiments and data analysis; Z.Z. and C.W. performed computational simulations; C.W., Z.Z, and X.S.Z. wrote the paper; all authors edited the paper; X.S.Z. acquired funding.

## Supporting information

Supporting Information

Supplemental Movie 1

Supplemental Movie 2

Supplemental Movie 3

Supplemental Movie 4

Supplemental Movie 5

## Data Availability

The data that support the findings of this study are available in the paper and/or the Supporting Information of this article.
