# Supplementary material for: Wireless Magnetic Robot for Precise Hierarchical Control of Tissue Deformation
Source: Adv Sci (Weinh). 2024 Jul 23;11(35):2308619. doi: 10.1002/advs.202308619 (PMC11425225; doi:10.1002/advs.202308619)
Supplement: Supplementary file 1 — Supporting Information [file ADVS-11-2308619-s004.pdf]

Supporting Information for
Wireless Magnetic Robot for Precise Hierarchical Control of Tissue Deformation

Chao Wang, Zhi Zhao, Joonsu Han, Arvin Ardebili Sharma, Hua Wang, Xiaojia Shelly Zhang*

This PDF file includes:
Supplementary Text
Figs. S1 to S5
Legends for Movies S1 to S5
References
Other Supporting Information for this manuscript include the following:
Movies S1 to S5

*Corresponding author.
Email address: zhangxs@illinois.edu (Xiaojia Shelly Zhang)

Supplementary Text

1. Constitutive models and finite element simulation

1.1. Constitutive model for magnetic robots

Let us consider a deformable solid occupying a domain Ω with the displacement boundary condition $\boldsymbol{u} = \overline{\boldsymbol{u}}$ on $\Gamma_{\boldsymbol{u}}$ and traction boundary condition $\boldsymbol{t} = \bar{\boldsymbol{t}}$ on $\Gamma_{\boldsymbol{t}}$, such that $\Gamma_{\boldsymbol{u}} \cup \Gamma_{\boldsymbol{t}} = \partial\Omega$ and $\Gamma_{\boldsymbol{u}} \cap \Gamma_{\boldsymbol{t}} = \emptyset$. In the absence of mechanical body force, we denote $\boldsymbol{x}$ and $\boldsymbol{X}$ as the position vectors in current and reference configurations, respectively. The deformation of the solid is characterized by a mapping function $\boldsymbol{x} = \chi(\boldsymbol{X})$. The displacement field $\boldsymbol{u}$ links the two deformation configurations through $\boldsymbol{x} = \boldsymbol{X} + \boldsymbol{u}$. Accordingly, the deformation gradient is defined as $\boldsymbol{F} = \frac{\partial \boldsymbol{x}}{\partial \boldsymbol{X}}$.

The magnetic robots are assumed to be ideal hard-magnetic soft materials and can be modeled via a constitutive model developed by [1]. This model has been validated to possess prediction in good agreement with experimental results [1]. The total Helmholtz free energy of magnetic robot per unit volume W_R in the reference configuration can be expressed as

$$W_R(\boldsymbol{F}) = W_E(\boldsymbol{F}) + W_M(\boldsymbol{F}) = W_E(\boldsymbol{F}) - \frac{1}{\mu_0}\big(\boldsymbol{F}\boldsymbol{B}_r\big) \cdot \boldsymbol{B}_a, \tag{1}$$

where $W_E(\boldsymbol{F})$ is the hyperelastic stored-energy function for characterizing the nonlinear elasticity of the soft material; $W_M(\boldsymbol{F})$ represents the magnetic potential energy; $\boldsymbol{F}$ is the deformation gradient; $\mu_0 = 1.257 \times 10^{-6}$ H/m is the vacuum (or air) magnetic permeability; $\boldsymbol{B}_r$ is the residual magnetic flux density in the reference configuration; and $\boldsymbol{B}_a$ is the applied magnetic flux density, which is assumed to remain uniform and unchanged. In this work, $W_E(\boldsymbol{F})$ is taken to be a I_1-based model [2] given by

$$W_E(\boldsymbol{F}) = \sum_{i=1}^{2} \frac{3^{1-\alpha_i}}{2\alpha_i}\mu_i \left(I_1^{\alpha_i} - 3^{\alpha_i}\right) - \sum_{i=1}^{2} \mu_i \ln J + \frac{\mu'}{2}(J-1)^2, \tag{2}$$

where $I_1 = \operatorname{tr}(\boldsymbol{C})$ is the first invariant of the right Cauchy–Green deformation tensor $\boldsymbol{C} = \boldsymbol{F}^{\mathrm{T}}\boldsymbol{F}$; J is the determinant of $\boldsymbol{F}$; α_i $(i = 1, 2)$ are real-valued material parameters; μ' and $\mu = \mu_1 + \mu_2$ are the first and second Lamé constant under the initial state, respectively. Note that μ_1, μ_2, α_1, α_2 are material constants and need to be experimentally characterized. The last two terms in the expression describe compressibility. In the context of 2D simulation (plane stress), these terms are omitted in this study, transforming the model into an incompressible one.

1.2. Constitutive model for bio-tissues

In this study, we consider the bio-tissues to be isotropic and homogeneous. We employ the Ogden model [3], which has gained widespread popularity as a constitutive model in soft tissue biomechanics, to model the nonlinearity of the biotissues. The stored-energy function is expressed as:

$$W_T(\lambda_1, \lambda_2, \lambda_3) = \sum_{p=1}^{2} \frac{\mu_p}{\alpha_p}\left(\lambda_1^{\alpha_p} + \lambda_2^{\alpha_p} + \lambda_3^{\alpha_p} - 3\right) - \sum_{i=1}^{2} \mu_i \ln J + \frac{\mu'}{2}(J-1)^2, \tag{3}$$

where λ_1, λ_2, λ_3 are the principal stretches depending on deformation gradient $\boldsymbol{F}$; μ_1, μ_2, α_1, α_2 are material constants that need to be experimentally characterized. In the context of 2D simulation (plane stress), the last two terms are omitted in this study, transforming the model into an incompressible one.

1.3. Constitutive model for connectors

To model the connector material (elastomer) in 2D that is used for performance tests of the wireless magnetic robots, we again use the incompressible I_1-based model [2] given in (2):

$$W_C(\boldsymbol{F}) = \sum_{i=1}^{2} \frac{3^{1-\alpha_i}}{2\alpha_i}\mu_i \left(I_1^{\alpha_i} - 3^{\alpha_i}\right), \tag{4}$$

where the definitions of the variables and constants are the same as before.

1.4. Finite element simulation

We use finite element method to simulate the magneto-mechanical performance of the robots and the underlying tissues. Adopting a total Lagrangian formulation and neglecting traction and body force, we formulate a displacement-based finite element problem with the total potential energy given by

$$\Pi(\boldsymbol{u}^{(\ell)}) = \sum_{e} \left(W_{E,e}(\boldsymbol{u}_e^{(\ell)}) + W_{M,e}(\boldsymbol{u}_e^{(\ell)}) + W_{T,e}(\boldsymbol{u}_e^{(\ell)}) \right), \tag{5}$$

where $\boldsymbol{u}_e^{(\ell)}$ is the elementwise displacement field under the applied magnetic field $\boldsymbol{B}_a^{(\ell)}$, $W_{E,e}$ and $W_{M,e}$ represent the elementwise stored energy for the matrix material and magnetic energy for the magnetic robot, respectively, and $W_{T,e}$ denotes the elementwise stored energy for the tissue (or connector). The detailed models adopted are elaborated in Section S1.

Minimizing the total potential energy $\Pi(\boldsymbol{u}^{(\ell)})$ with respect to the global displacement field $\boldsymbol{u}$ gives the discretized stationary condition

$$\mathbf{R}(\boldsymbol{u}^{(\ell)}) = \frac{\partial \Pi}{\partial \boldsymbol{u}^{(\ell)}}(\boldsymbol{u}^{(\ell)}) = \mathbf{F}_{\text{int}}(\boldsymbol{u}^{(\ell)}) = \mathbf{0}, \tag{6}$$

which governs the equilibrium of the discretized system. We refer to $\mathbf{R}$ and $\mathbf{F}_{\text{int}}$ as the global residual vector and global internal force vectors, respectively. In this work, the nonlinear equation (6) is solved using the Newton-Raphson method [4] with the inexact line search method [5, 6]. This nonlinear solver makes use of consistently linearized tangent stiffness matrices, for which we apply the "`fsparse`" routine [7, 8] to enhance the computational efficiency of sparse matrix assembly. When simulating the 3D problem, we employ reduced and selective integration [9] to address the locking issue, assuming a Poisson's ratio of 0.49 for both magnetic robots and tissues.

2. Topology optimization framework

Our design goal is to optimize the magnetic robots (both geometry and magnetization distributions) to achieve target deformation modes on tissues. We first present a design space parameterization scheme that simultaneously parametrizes matrix material topology and remnant magnetization distribution. Then, we present how to use the parameterized variables to interpolate the energy functions ((1), (3), (4)) to characterize the magneto-mechanical interaction between hard-magnetic soft material and tissues (or connectors). We briefly describe the finite element simulation for numerically computing the magneto-mechanical performance. Lastly, we report the established optimization formulation for optimizing magnetic robots.

2.1. Design parametrization of magnetic robot

Parameterization of matrix distribution (i.e., topology). The distribution of matrix characterizes the geometry of the biomaterial. Here, a density-based approach [10] is adopted. The matrix geometry is described and associated with the density variable $\boldsymbol{\rho}$ with ρ_e for the eth element. We apply the Heaviside projection operator [11] (with 1/2 being its threshold) to the density variable, obtaining the physical density variables $\overline{\boldsymbol{\rho}}$ with $\overline{\rho}_e$ given by

$$\overline{\rho}_e = \frac{\tanh(\frac{\beta_\rho}{2}) + \tanh(\beta_\rho(\widetilde{\rho}_e - \frac{1}{2}))}{2\tanh(\frac{\beta_\rho}{2})}, \tag{7}$$

with β_ρ being the parameter controlling the discreteness of the projection, and $\widetilde{\rho}_e$ being the intermediate design variable regularized via the density filter [12, 13] as follows:

$$\widetilde{\rho}_e = \frac{\sum_{i\in\mathcal{I}_e(R_\rho)} w_\rho^{(i,e)} v_i \rho_i}{\sum_{i\in\mathcal{I}_e(R_\rho)} w_\rho^{(i,e)} v_i}, \tag{8}$$

where $\mathcal{I}_e(R_\rho)$ is the eth element set within a prescribed region defined by a circle with a radius of R_ρ at the centroid of eth element; and v_i is the ith element volume. The weighting factor $w_\rho^{(i,e)}(R_\rho, q_\rho)$ depends on

the distance between the centroids of ith and eth elements (denoted as $\boldsymbol{X}_i$ and $\boldsymbol{X}_e$, respectively), namely, $w_\rho^{(i,e)} = 1 - (\|\boldsymbol{X}_i - \boldsymbol{X}_e\| / R_\rho)^{q_\rho}$, with q_ρ being the power of the filter. The physical design variable $\overline{\rho}_e$ serves as an indicator of whether a given location in space is solid or void: $\overline{\rho}_e = 1$ represents solid and $\overline{\rho}_e = 0$ represents void.

Parameterization of magnetization distribution. The residual magnetic flux density at each location of the design is selected from a set of N_m pre-selected candidate residual magnetic flux densities, $\boldsymbol{B}_r^{(1)}$,...,$\boldsymbol{B}_r^{(N_m)}$, each pointing at one direction. Formally, we define the residual magnetic flux density in element e as

$$\boldsymbol{B}_{r,e} = \sum_{j=1}^{N_m} \left(\overline{m}_e^{(j)}\right)^{p_m} \boldsymbol{B}_r^{(j)}. \tag{9}$$

In the above interpolation, $\overline{m}_e^{(j)}$ is the physical magnetization variable which serves as an indicator of the magnetization of element e: $\overline{m}_e^{(j)} = 1$ means the jth candidate residual magnetic flux density $\boldsymbol{B}_r^{(j)}$ is selected, and $\overline{m}_e^{(j)} = 0$ means the jth candidate residual magnetic flux density $\boldsymbol{B}_r^{(j)}$ is not selected. A Solid Isotropic Material with Penalization (SIMP)-type [14, 15] penalization power p_m is introduced to penalize the mixture of candidate magnetizations and to promote the convergence of the physical magnetization variables $\overline{m}_e^{(j)}$ to either 1 or 0.

In this work, we aim to promote discrete magnetization distribution and allow non-magnetized regions to appear in the design. we adopt the Hypercube-to-Simplex Projection (HSP) approach [16, 17] to define $\overline{m}_e^{(j)}$. By using HSP, the physical magnetization variables can automatically satisfy the following two constraints: 1) $\sum_{j=1}^{N_m} \overline{m}_e^{(j)} \leq 1$ and 2) $\overline{m}_e^{(j)} \geq 0$, $\forall j$. The HSP approach defines the physical magnetization variables $\overline{m}_e^{(j)}$ as

$$\overline{m}_e^{(j)} = \sum_{i=1}^{2^{N_m}} s_i^{(j)} \left((-1)^{\left(N_m + \sum_{j=1}^{N_m} c_i^{(j)}\right)} \prod_{k=1}^{N_m} \left(\overline{\xi}_e^{(k)} + c_i^{(k)} - 1\right)\right), \tag{10}$$

where $\overline{\xi}_e^{(j)}$ is the magnetization variable subject to filtering and projection through the same expressions in (7) and (8). The parameter $c_i^{(j)} = \{0, 1\}$ is the ith vertex of an N_m-dimensional unit hypercube for the jth candidate remnant magnetization vector, and $s_i^{(j)}$ is the mapped vertex of an N_m-dimensional standard simplex domain:

$$s_i^{(j)} = \begin{cases} \dfrac{c_i^{(j)}}{\sum_{j=1}^{N_m} c_i^{(j)}} & \text{if } \sum_{j=1}^{N_m} c_i^{(j)} \geq 1, \\ 0 & \text{otherwise.} \end{cases} \tag{11}$$

2.2. Interpolation of the energy function

To describe the nonlinear magneto-mechanical behavior of magnetic robot and tissues (or connectors), we introduce the following interpolation of the energy function from the physical variables $\overline{\boldsymbol{\rho}}$ and $\overline{\boldsymbol{m}}^{(j)}$, $j = 1, ..., N_m$. The interpolated element-wise energy $W_e^{(\ell)}$ is given by

$$W_e^{(\ell)}\left(\overline{\rho}_e, \overline{m}_e^{(1)}, ..., \overline{m}_e^{(N_m)}, \boldsymbol{u}_e^{(\ell)}\right) = \\ \left[\epsilon + (1-\epsilon)\left(\overline{\rho}_e\right)^{p_\rho}\right] W_{E,e}\left(\boldsymbol{u}_e^{(\ell)}\right) + \left(\overline{\rho}_e\right)^{p_\rho} W_{M,e}\left(\boldsymbol{u}_e^{(\ell)}, \boldsymbol{B}_{r,e}(\overline{m}_e^{(1)}, ..., \overline{m}_e^{(N_m)})\right) + W_{T,e}\left(\boldsymbol{u}_e^{(\ell)}\right), \tag{12}$$

where $\boldsymbol{u}_e^{(\ell)}$ is the displacement vector in element e under the l-th applied magnetic field $B_a^{(\ell)}$; and $\epsilon = 10^{-5}$ is a small value to avoid singular stiffness. In the above interpolation formula, the SIMP approach [10, 15] is used to penalize both elastic-stored energy and magnetic free energy to promote a discrete design. The penalization parameters p_ρ associated with both energies are taken to be the same. Based on our numerical experience, excessive deformations of low-stiffness regions can lead to numerical instabilities during the optimization. Thus, an energy interpolation scheme [18] is applied to the stored-energy W_E to address the

numerical instabilities of low stiffness regions (defined to be regions with $(\overline{\rho}_e)^{p_\rho} \leq 0.01$). We also apply the same concept to the magnetic free energy W_M so that the magnetic actuation in low-stiffness regions is negligible. The symbol W_T refers to the stored energy of tissues. They are not in the design space, thus their stored energy is not associated with design variables.

2.3. Topology optimization formulation

With the introduced design space parameterization and free-energy interpolation schemes, we now present the topology optimization formulation to generate the magneto-active biorobots. The mesh Ω_h is composed of N_e elements and N_n nodes. The goal of the topology optimization is to maximize the tissue displacements at control points with the constraints and the nested equilibrium satisfied. Formally, we formulate the topology optimization problem as:

$$\begin{aligned}
&\min_{\boldsymbol{\rho},\boldsymbol{\xi}^{(1)},\dots,\boldsymbol{\xi}^{(N_m)}} \quad \max_{\substack{\ell \in \{1,\dots,N_\ell\} \\ \alpha \in \{1,\dots,N_\alpha^{(\ell)}\}}} \quad u_\alpha^{(\ell)}, \\
&\text{s.t.:} \qquad \frac{\boldsymbol{v}^T\overline{\boldsymbol{\rho}}}{|\Omega_h|} \leq v_{\max}, \\
&\qquad\qquad \left\{ \sum_{e=1}^{N_e} \left[\frac{w_\sigma(\overline{\rho}_e)}{v_e} \int_{\Omega_{h,e}} \sigma_{\mathrm{VM}}\Big(\boldsymbol{\sigma}_E\big(\boldsymbol{u}^{(\ell)}\big)\Big) \mathrm{d}\boldsymbol{X} \right]^{p_n} \right\}^{1/p_n} \leq \sigma_{\max}^{(\ell)}, \quad \ell = 1,\dots,N_\ell, \\
&\qquad\qquad \mathbf{R}(\overline{\boldsymbol{\rho}}, \overline{\boldsymbol{m}}^{(1)},\dots,\overline{\boldsymbol{m}}^{(N_m)}, \boldsymbol{u}^{(\ell)}) = \mathbf{0}, \quad \ell = 1,\dots,N_\ell, \\
&\qquad\qquad \mathbf{0} \leq \boldsymbol{\rho} \leq \mathbf{1}, \\
&\qquad\qquad \mathbf{0} \leq \boldsymbol{\xi}^{(j)} \leq \mathbf{1}, \quad j = 1,\dots,N_m,
\end{aligned} \tag{13}$$

where $u_\alpha^{(\ell)}$ is the actual displacement at the α-th control degree of freedom (DOF) of the underlying tissue under applied magnetic field $\boldsymbol{B}_a^{(\ell)}$; $\boldsymbol{v}$ is a vector collecting element volumes with its eth component v_e being the volume of element e, and $v_{\max}$ is the prescribed maximum volume fraction. A min-max formulation [19] is employed to maximize the displacement at the control point, with the appropriate sign for the desired deformation modes. To eliminate thin members and limit excessive local deformations in optimized designs, we introduce an aggregated von Mises stress constraint [20, 21, 22, 23, 24] (using the p-norm approximation with the factor p_n) for each applied magnetic field. The element-level von Mises stress σ_{VM} is computed from the mechanical part of the Cauchy stress σ_E. The stress relaxation approach [22] is adopted giving $w_\sigma(\overline{\rho}_e) \doteq \epsilon + (1-\epsilon)\overline{\rho}_e^q$ with q being 1/2.

The proposed formulation (13) is solved by a gradient-based method of moving asymptotes (MMA) [25]. The sensitivities information of objective and constraint functions with respect to the design variables are derived by the adjoint method [10].

3. Fabrication and characterization of biomaterials and tissues

3.1. Characterization of the biological tissues

In this study, we conduct uniaxial tensile and compressive tests on porcine skeletal muscle, liver, and myocardium tissue samples. To ensure consistent sample dimensions, 3D-printed cutting guides (for tension: 61 mm × 12 mm × 5 mm; for compression: 10 mm × 10 mm × 10 mm) are used to cut the samples into the desired shape. After the tissue strips were prepared, uniaxial tension and compression experiments are performed at room temperature. The experiments are conducted using a loading machine (Instron 68TM-30). The prepared samples and specific setups are illustrated in Figs. S1A-C. For the uniaxial tension experiments, the tissue samples are fixed in clamps covered with sandpaper from the inside to avoid tissue slippage. Tension experiments are carried out until the sample failed. For performing the compression experiments, tissue samples are positioned between two "T"-shape steel loading bars. Before placing the samples between the loading bars, they are sprayed with silicon oil to avoid friction. All the tests are performed at a strain rate of 0.5%/s. The force data F is directly obtained from the loading

machine, and the nominal stress data is calculated by $P = F/A$, where A is the cross-sectional area of the undeformed tissue sample, which is taken as the averaged value measured at 3 three locations. For the tensile testing, to get the strain data, we spray speckle patterns on the surface of the tissue samples. The loading history is video-recorded using a commercial camera (SONY α7R), and images are sampled according to the frequency of the force. Digital image correlation (DIC) analysis is performed using the Matlab toolbox "ncorr" to obtain the displacement field, from which strain is calculated based on the displacement of an extensometer line within the range of uniform strain. For the compressive testing, the strain information is directly obtained from the loading machine. Figures S1D-F show the measured stress-strain curves and the corresponding fitted curves using the Ogden model in Eq. 3 for the three types of tissues.

3.2. Characterization of the biomaterial

The biomaterials investigated in this study include the hard magnetic soft material (HMSM) made of PDMS elastomer (20:1 base-to-agent ratio) with 0 vol% (pure PDMS), 15 vol%, and 25 vol% NdFeB magnetic particles, and Eco-flex 00-30 used to make connectors for the robots' performance tests. The mechanical properties of these biomaterials are determined by fitting the parameters in the constitutive model Eq. 2 to the experimentally obtained uniaxial stress-strain relationships. We fabricate the compression and tension testing samples following the standard [26, 27] as shown in Figs. S2A-B. The results for the measured and fitted stress-strain curves for the biomaterials are presented in Figs. S2C-F. The fitted material constants (α_1, μ_1, α_2, μ_2) are given in the figures.

3.3. Experimental procedure for DIC

We employed the DIC approach to capture the full-field displacement of the tissue during magnetic actuation. Initially, speckle patterns were applied to the tissue surface using RUST-OLEUM spray paint, featuring a diameter size of 3-5 pixels. A SONY α7R camera with the FE 24-70mm F2.8 GM II Lens was positioned appropriately to record the actuation process from the undeformed to the deformed state at a frame rate of 29.97 frames/second. Note that since the magnetic actuation is quite fast and the induced tissue strain is relatively larger, to ensure the effectiveness and accuracy of the DIC analyzed results, we extract every frame between the initial undeformed and the final deformed frames from the recorded video. Subsequently, these extracted images underwent batch processing in Photoshop to accommodate a better field-of-view and adjust pattern contrast. For instance, in the shearing mode analysis, the processed image resolution is of 2166 $\times$ 2160 pixels. The images were then imported to the open source Ncorr package [28] in Matlab to calculate the displacement fields. The areas covered by the robots were excluded from the region-of-interest. The resulting displacement fields have a resolution of 0.0288 mm/pixel. A subset with radius of 1.786 mm and spacing of 0.144 mm was chosen. To accommodate large displacements, the high-strain analysis feature was enabled. The correlation algorithm consistently updated the reference image, and the analysis was executed in a backwards manner to appropriately handle the rapid deformation. In this process, the final deformed image served as the reference, and all subsequent correlations were conducted in relation to it.

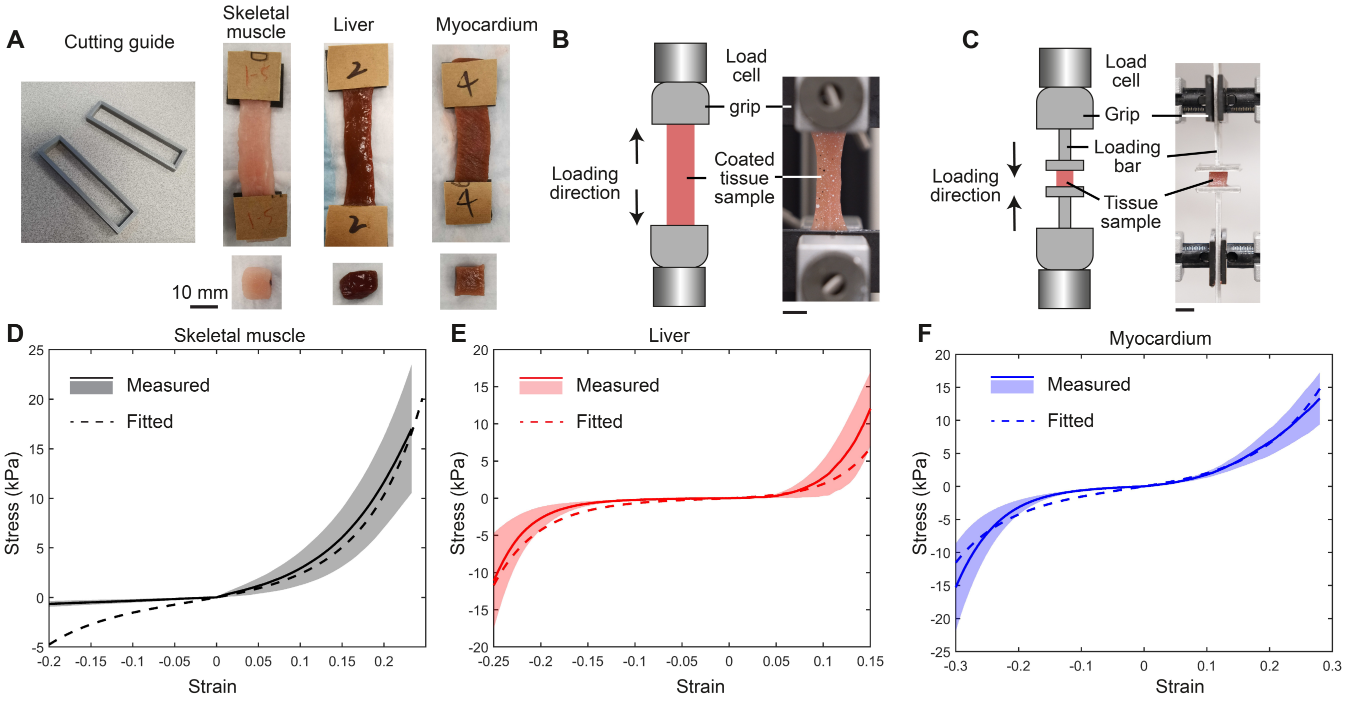

Figure S1: Biological tissue characterization. A) Uniaxial tensile and compressive testing tissue samples: skeletal muscle (n = 12 for tension; n = 12 for compression), liver (n = 6 for tension; n = 6 for compression), and myocardium (n = 6 for tension; n = 6 for compression). B,C) Testing setup for the B) uniaxial tensile and C) compressive loading. D-F) Measured and fitted stress-strain curves for the D) skeletal muscle, E) liver, and F) myocardium tissues. Scale bar: 10 mm.

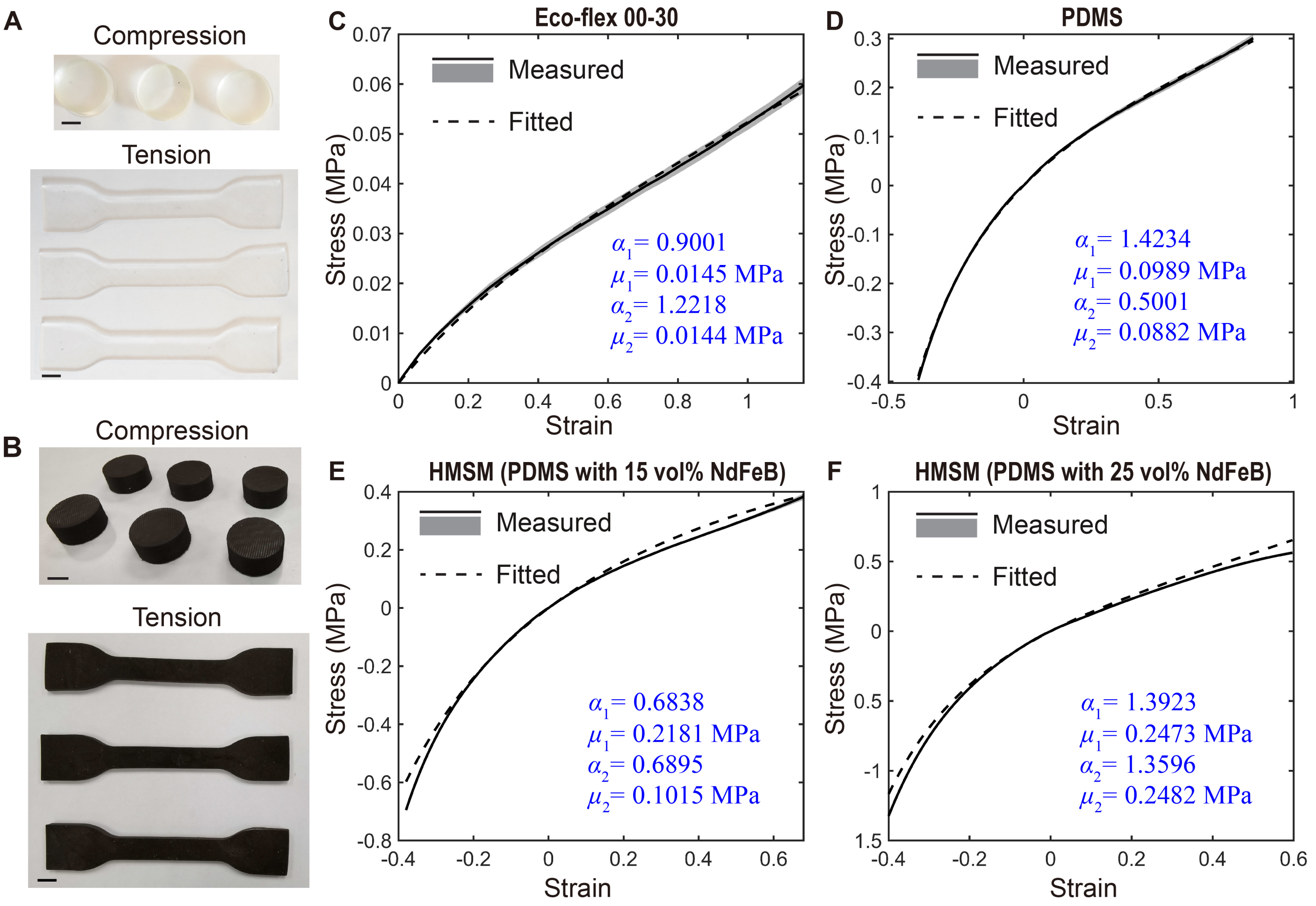

Figure S2: Biomaterial characterization. A,B) Selective compression and tension samples for A) pure PDMS (20:1 base-to-agent ratio) and B) hard magnetic soft materials. C-F) Characterized stress-strain curves for Eco-flex 00-30 (n = 7), PDMS (20:1 base-to-agent ratio, n = 3), and HMSM with 15 vol% (n = 3) and 25 vol% (n = 3) NdFeB magnetic particles, respectively. Scale bar: 10 mm.

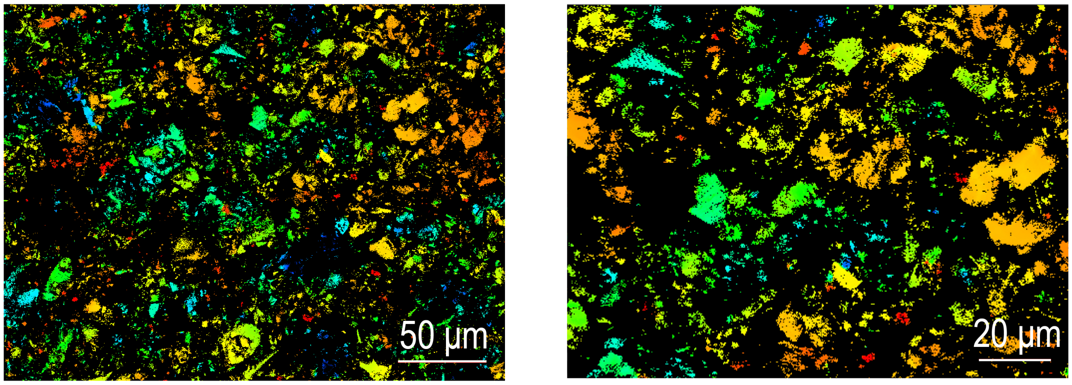

Figure S3: Optical microscopic images of a hard magnetic soft material with 25 vol% NdFeB particles at two magnifications through a thickness of 60 μm. Different colors in the images signify the location of particles along the thickness direction.

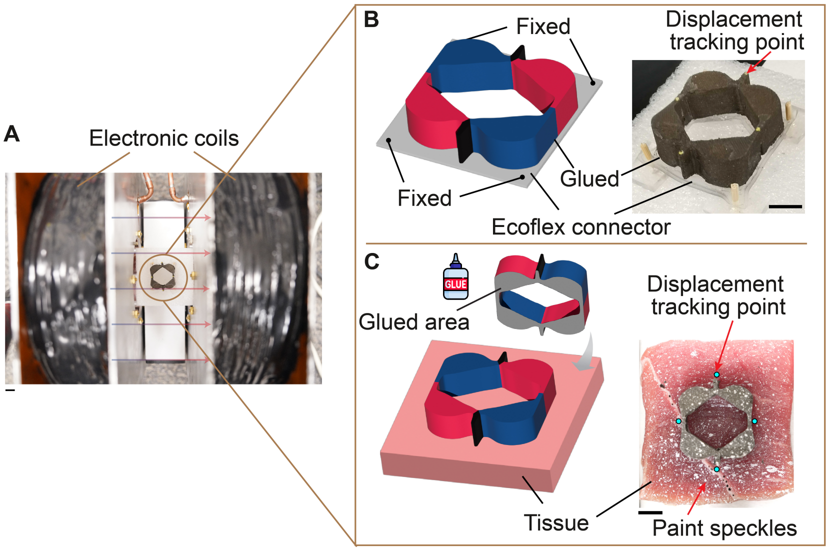

Figure S4: Experiment setup. A) Experiment setup with the magnetic actuation. B) Illustration of the setup for the robot performance tests. C) Illustration of the setup for the *ex vivo* experiment. Scale bar: 10 mm.

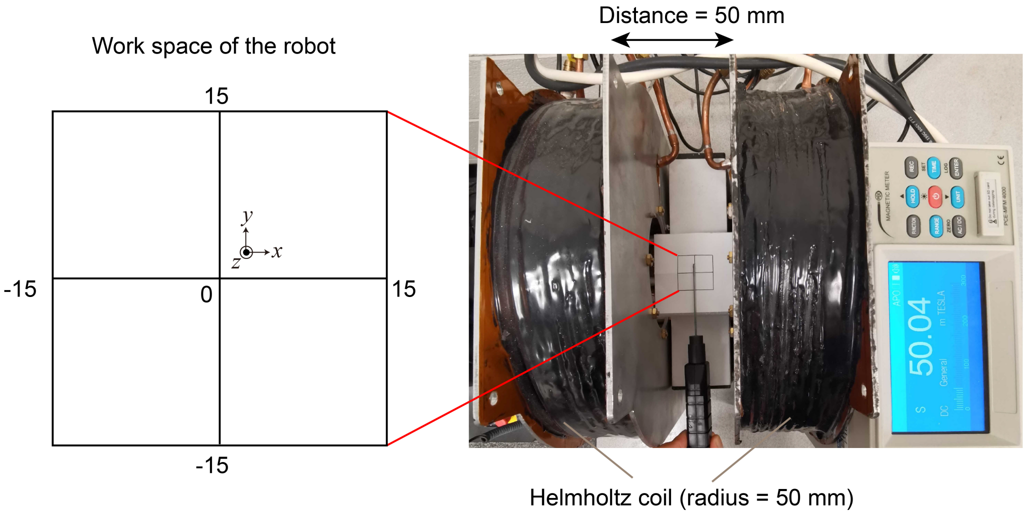

Figure S5: Measurement of the magnetic field generated by the Helmholtz coil within the workspace (30 mm × 30 mm): The measured Ba is 50.04 ± 0.06 mT.

Movie S1. Workflow of wireless magnetic robot.
Movie S2. Wireless magnetic robots with uniaxial motion.
Movie S3. Wireless magnetic robots with shearing motion.
Movie S4. Wireless magnetic robots with dual-mode motion.
Movie S5. Wireless magnetic robots-induced biaxial motion of liver and myocardium tissues.

References

[1] R. Zhao, Y. Kim, S. A. Chester, P. Sharma, X. Zhao, Mechanics of hard-magnetic soft materials, Journal of the Mechanics and Physics of Solids 124 (2019) 244–263.

[2] O. Lopez-Pamies, A new I1-based hyperelastic model for rubber elastic materials, Comptes Rendus Mecanique 338 (1) (2010) 3–11.

[3] M. J. Lohr, G. P. Sugerman, S. Kakaletsis, E. Lejeune, M. K. Rausch, An introduction to the ogden model in biomechanics: benefits, implementation tools and limitations, Philosophical Transactions of the Royal Society A 380 (2234) (2022) 20210365.

[4] Nonlinear finite elements for continua and structures, John wiley & sons, 2014.

[5] L. Armijo, Minimization of functions having lipschitz continuous first partial derivatives, Pacific Journal of Mathematics 16 (1) (1966) 1–3.

[6] X. Zhang, A. S. Ramos, G. H. Paulino, Material nonlinear topology optimization using the ground structure method with a discrete filtering scheme, Structural and Multidisciplinary Optimization 55 (6) (2017) 2045–2072.

[7] F. Ferrari, O. Sigmund, A new generation 99 line matlab code for compliance topology optimization and its extension to 3d, Structural and Multidisciplinary Optimization 62 (4) (2020) 2211–2228.

[8] S. Engblom, D. Lukarski, Fast matlab compatible sparse assembly on multicore computers, Parallel Computing 56 (2016) 1–17.

[9] D. S. Malkus, T. J. Hughes, Mixed finite element methods—reduced and selective integration techniques: a unification of concepts, Computer Methods in Applied Mechanics and Engineering 15 (1) (1978) 63–81.

[10] Topology optimization: theory, methods, and applications, Springer Science & Business Media, 2013.

[11] F. Wang, B. S. Lazarov, O. Sigmund, On projection methods, convergence and robust formulations in topology optimization, Structural and Multidisciplinary Optimization 43 (6) (2011) 767–784.

[12] B. Bourdin, Filters in topology optimization, International Journal for Numerical Methods in Engineering 50 (9) (2001) 2143–2158.

[13] O. Sigmund, Morphology-based black and white filters for topology optimization, Structural and Multidisciplinary Optimization 33 (4-5) (2007) 401–424.

[14] G. I. Rozvany, M. Zhou, T. Birker, Generalized shape optimization without homogenization, Structural Optimization 4 (3-4) (1992) 250–252.

[15] M. P. Bendsøe, Optimal shape design as a material distribution problem, Structural Optimization 1 (4) (1989) 193–202.

[16] Y. Zhou, T. Nomura, K. Saitou, Multi-component topology and material orientation design of composite structures (MTO-C), Computer Methods in Applied Mechanics and Engineering 342 (2018) 438–457.

[17] X. S. Zhang, H. Chi, Z. Zhao, Topology optimization of hyperelastic structures with anisotropic fiber reinforcement under large deformations, Computer Methods in Applied Mechanics and Engineering 378 (2021) 113496.

[18] F. Wang, B. S. Lazarov, O. Sigmund, J. S. Jensen, Interpolation scheme for fictitious domain techniques and topology optimization of finite strain elastic problems, Computer Methods in Applied Mechanics and Engineering 276 (2014) 453–472.

[19] N. Olhoff, Multicriterion structural optimization via bound formulation and mathematical programming, Structural Optimization 1 (1) (1989) 11–17.

[20] G. Cheng, Z. Jiang, Study on topology optimization with stress constraints, Engineering Optimization 20 (2) (1992) 129–148.

[21] G. Cheng, X. Guo, ε-relaxed approach in structural topology optimization, Structural Optimization 13 (4) (1997) 258–266.

[22] M. Bruggi, On an alternative approach to stress constraints relaxation in topology optimization, Structural and Multidisciplinary Optimization 36 (2) (2008) 125–141.

[23] P. Duysinx, M. P. Bendsøe, Topology optimization of continuum structures with local stress constraints, International Journal for Numerical Methods in Engineering 43 (8) (1998) 1453–1478.

[24] Z. Zhao, X. S. Zhang, Topology optimization of hard-magnetic soft materials, Journal of the Mechanics and Physics of Solids 158 (2022) 104628.

[25] K. Svanberg, The method of moving asymptotes—a new method for structural optimization, International Journal for Numerical Methods in Engineering 24 (2) (1987) 359–373.

[26] ASTM International, ASTM D412-16: Standard test methods for vulcanized rubber and thermoplastic elastomers—tension (2016).

[27] ASTM International, ASTM D575-91, Standard test methods for rubber properties in compression (2018).

[28] J. Blaber, B. Adair, A. Antoniou, Ncorr: open-source 2d digital image correlation matlab software, Experimental Mechanics 55 (6) (2015) 1105–1122.
